# High Intensity Focused Ultrasound‐Driven Nanomotor for Effective Ferroptosis‐Immunotherapy of TNBC

**DOI:** 10.1002/advs.202305546

**Published:** 2024-02-11

**Authors:** Xiangrong Yu, Xuejing Li, Qingwang Chen, Siyu Wang, Ruizhe Xu, Ying He, Xifeng Qin, Jun Zhang, Wuli Yang, Leming Shi, Ligong Lu, Yuanting Zheng, Zhiqing Pang, Shaojun Peng

**Affiliations:** ^1^ Guangdong Provincial Key Laboratory of Tumor Interventional Diagnosis and Treatment Zhuhai People's Hospital (Zhuhai Hospital Affiliated with Jinan University) Zhuhai Guangdong 519000 P. R. China; ^2^ Key Laboratory of Smart Drug Delivery, School of Pharmacy Fudan University Shanghai 201203 P.R. China; ^3^ State Key Laboratory of Genetic Engineering, School of Life Sciences and Human Phenome Institute Fudan University 2005 Songhu Road Shanghai 200438 P.R. China; ^4^ Department of Radiology, Huashan Hospital, State Key Laboratory of Medical Neurobiology Fudan University 12 Wulumuqi Middle Road Shanghai 200040 China; ^5^ State Key Laboratory of Molecular Engineering of Polymers & Department of Macromolecular Science Fudan University Shanghai 200433 China

**Keywords:** ferroptosis, gambogic acid, high intensity focused ultrasound, immunotherapy, nanomotors, triple‐negative breast cancers

## Abstract

The heterogeneity of triple‐negative breast cancers (TNBC) remains challenging for various treatments. Ferroptosis, a recently identified form of cell death resulting from the unrestrained peroxidation of phospholipids, represents a potential vulnerability in TNBC. In this study, a high intensity focused ultrasound (HIFU)‐driven nanomotor is developed for effective therapy of TNBC through induction of ferroptosis. Through bioinformatics analysis of typical ferroptosis‐associated genes in the FUSCCTNBC dataset, gambogic acid is identified as a promising ferroptosis drug and loaded it into the nanomotor. It is found that the rapid motion of nanomotors propelled by HIFU significantly enhanced tumor accumulation and penetration. More importantly, HIFU not only actuated nanomotors to trigger effective ferroptosis of TNBC cells, but also drove nanomotors to activate ferroptosis‐mediated antitumor immunity in primary and metastatic TNBC models, resulting in effective tumor regression and prevention of metastases. Overall, HIFU‐driven nanomotors show great potential for ferroptosis‐immunotherapy of TNBC.

## Graphical abstract

Triple‐negative breast cancer (TNBC) poses a significant treatment challenge due to its heterogeneous nature. Researchers have developed a nanomotor powered by high intensity focused ultrasound (HIFU) to deliver a ferroptosis drug, which is identified through bioinformatics analysis. This HIFU‐responsive nanomotor is able to trigger ferroptosis, activate the immune system, reduce tumor growth, and prevent metastasis in TNBC models.

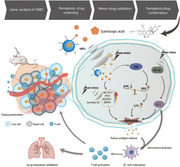

## 1 Introduction

The prevalence of breast cancer in women worldwide is on the rise in the majority of countries, resulting in the highest mortality rate among female cancer cases.^[^ ^1^ ^]^ Triple‐negative breast cancers (TNBC), characterized by the absence of estrogen receptor (ER), progesterone receptor (PR), and human epidermal growth factor receptor 2 (HER2) expression, are recognized as the most aggressive subtype of breast cancer, accounting for ≈15% of all cases.^[^ ^2^ ^]^ The aggressive progression of TNBC is attributed to its pathological and molecular heterogeneity, which ultimately results in unfavorable clinical outcomes and prognosis for affected patients.^[^ ^3^ ^]^ Symptom palliation is the primary purposeof local therapy modalities such as surgical resection and radiation, while neoadjuvant chemotherapy is generally effective in treating TNBC.^[^ ^2^ ^]^ However, patients with residual disease following this treatment approach exhibit significantly poorer prognosis and lower survival rates.^[^ ^4^ ^]^ The clinical efficacy of checkpoint inhibitors, such as pembrolizumab and atezolizumab, in combination with chemotherapy, is contingent upon the biomarker status and tumor microenvironment (TME) in TNBC.^[^ ^5^ ^]^ Metastatic TNBC demonstrates a diminished immune response compared to primary tumors, which dampens the optimism surrounding cancer immunotherapy.^[^ ^6^ ^]^ Consequently, these findings underscore the pressing necessity for innovative and more efficacious therapeutic approaches in the management of TNBC and its metastatic form.

​Ferroptosis, an iron‐dependent regulated necrosis that allows discrimination from other non‐apoptotic modes of cell death, culminates in an unconstrained lipid peroxidation caused by metabolic dysfunction.^[^ ^7^ ^]^ The abnormality of iron homeostasis and the enhancement of antioxidant defenses in several cancer types highlight the potential value of ferroptosis in tumor inhibition strategies.^[^ ^8^ ^]^ Iron‐rich tumors such as breast cancer are particularly sensitive to ferroptosis‐targeted drugs, which can achieve superior efficacy.^[^ ^9^ ^]^ For instance, dihydroisotanshinone I and sorafenib, prompt ferroptosis by directly or indirectly inhibiting the activity of the gluathione peroxidase 4 (GPX4) system.^[^ ^10^ ^]^ Lapatinib, an FDA‐approved anticancer drug, causes dysfunction of iron metabolism and activation of lipid peroxidation in breast cancer cells in combination with siramesine.^[^ ^11^ ^]^ Several preclinical studies have also advocated that predisposing cancer cells to ferroptosis by targeting relevant metabolic pathways may be an effective therapeutic strategy to eliminate breast cancer cells.^[^ ^12^ ^]^ Immunotherapies have raised hopes of successful control of advanced and metastatic tumors that have subverted immune surveillance.^[^ ^13^ ^]^ As ferroptosis belongs to an immunogenic form of cell death and augments antitumor immune responses, ferroptosis inducers may improve the efficacy of immunotherapy and trigger greater antitumor effectiveness in TNBC.^[^ ^14^ ^]^ Therefore, it is of great significance and value to identify novel ferroptosis inducers that achieve targeted induction of TNBC ferroptosis for propelling precise treatment of TNBC.

The emergence of nanomedicines has ushered in a new era for drug delivery, enhancing the therapeutic indices of active pharmaceutical ingredients.^[^ ^15^ ^]^ It is noteworthy that limited accumulation and penetration of therapeutic drugs in target sites pose a major challenge for nanomedicines, with less than 1% of the administered nanoparticles able to reach solid tumors through the inherent enhanced permeability and retention (EPR) effect.^[^ ^16^ ^]^ Nanomotors, as nanoscale machines capable of converting energy into mechanical motion, offer promising opportunities to surmount the low delivery efficiency to tumors.^[^ ^17^ ^]^ In particular, ultrasound‐powered nanomotors have garnered significant attention in tumor drug delivery due to their widespread clinical use.^[^ ^18^ ^]^ High frequency sound waves within the MHz regime serve as powerful tools for particle manipulation within tumor tissues.^[^ ^18^ ^]^ Additionally, preliminary evidence suggests that high intensity focused ultrasound (HIFU) may induce immune responses in cancers by destroying cancer cells, releasing tumor‐specific antigens, and promoting dendritic cell maturation.^[^ ^19^ ^]^ Although the underlying mechanism behind HIFU‐triggered cell death remains unknown, the shear stress induced by HIFU—similar to that generated by blood flow—may trigger expression of ferroptosis‐associated genes including heme oxygenase 1 (HOX1), glutathione S‐transferase (GST), sequestosome (SQSTM) etc., sensitizing tumor cells to ferroptosis‐inducers.^[^ ^20^ ^]^ Therefore, exploring HIFU‐driven nanomotors for targeted delivery of ferroptosis inducers deep into tumor tissues represents an ideal strategy for enhancing tumor ferroptosis and subsequently improving antitumor immunity.

**Figure 1:**
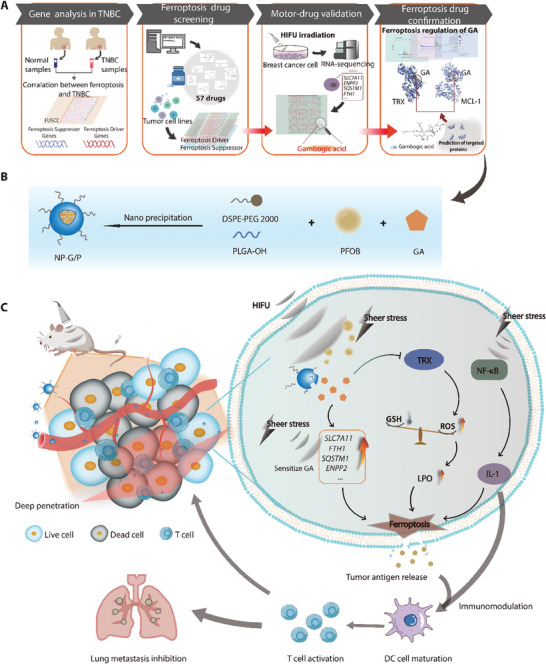
Screening of the ferroptosis inducer for TNBC therapy, and the preparation and antitumor mechanism of HIFU‐propelled nanomotors. A) The workflow to screen and identify the new ferroptosis inducer, gambogic acid, for TNBC treatment. B) The synthetic procedure of nanomotors (NP‐G/P). (C) The antitumor mechanism of HIFU‐driven nanomotors on TNBC through improved tumor penetration and robust ferroptosis‐immunotherapy.

In this study, a HIFU‐driven nanomotor (NP‐G/P) based on PEGylated poly (lactic‐co‐glycolic acid) (PLGA) nanoparticles (NPs) was developed for effective therapy of TNBC by inducing ferroptosis (**Figure** **1**). Perfluorooctyl bromide (PFOB) was loaded into the NPs to endow responsiveness to HIFU forces for propulsion and on‐demand drug release, while HIFU was utilized to sensitize TNBC cells to ferroptosis inducers through regulation of ferroptosis‐associated gene expression and modulation of the tumor immune microenvironment (Figure 1). First, novel ferroptosis drugs were identified through bioinformatics analysis of typical ferroptosis‐associated genes in the FUSCCTNBC datasets and chosen to be loaded into nanomotors to induce TNBC ferroptosis. The enhanced tumor accumulation and penetration pattern of nanomotors driven by HIFU were then investigated. Additionally, the effect and unique mechanisms of HIFU in actuating nanomotors to trigger effective ferroptosis in TNBC cells and boost ferroptosis‐mediated antitumor immunity in primary and metastatic TNBC models were carefully explored. Finally, we assessed the antitumor efficacy of this strategy using HIFU‐driven nanomotors for inducing ferroptotic cell death combined with immunotherapy against refractory tumors such as TNBC.

## 2 Results

### 2.1 Exploration of Ferroptosis Inducers Across TNBC Cohorts

**Figure 2:**
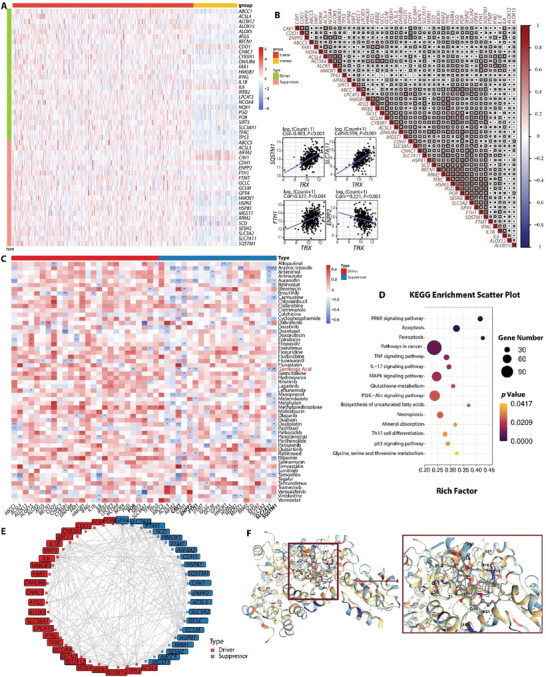
A) The heat map of differentially expressed ferroptosis regulator genes in TNBC and normal breast tissues. B) Diagrams of the correlations between the expression levels of ferroptosis regulators. The scatter plot represented the correlation between TXN (or TRX) and SQSTM1, SLC7A11, FTH1, and ENPP2. (C) Correlation between IC50 values of 57 predicted ferroptosis inducers and the expression levels of ferroptosis suppressors and drivers. *P < 0.05, **P < 0.01, and ***P < 0.001. D) The KEGG enrichment analysis of gene expressions in gambogic acid (GA)‐treated 4T1 cells compared with that in PBS‐treated 4T1 cells. (E) Protein‐protein interaction (PPI) network of ferroptosis regulator genes. F) Structures of the TRX and GA combination predicted with molecular docking.

To understand the correlation between ferroptosis and TNBC, we first summarized ferroptosis regulators by kyoto encyclopedia of genes and genomes (KEGG) pathway enrichment and ferroptosis gene libraries and selected 47 typical ferroptosis‐associated genes (26 driver genes and 21 suppressor genes), and analyzed the expression of these genes across TNBC and normal breast tissues from the FUSCCTNBC cohort. It was found that these genes exhibited marked differential expression in TNBC compared to adjacent normal tissues, with the majority showing significant upregulation (**Figure** **2**A; Figure S1, Supporting Information). Gene Ontology (GO) functional enrichment analysis was employed to further predict the signaling pathways associated with these genes. Ferroptosis and other processes including glutathione transport, response to metal ion, and oxidative stress response were highly enriched, indicating their positive relationship with ferroptosis‐related processes (Figure S2, Supporting Information). Correlation diagrams and protein‐protein interaction network were determined to clarify the transcriptome characteristics of ferroptosis regulators, which presented prevalent interactions among 47 ferroptosis regulators in TNBC (Figure 2B,E). These results suggest that targeting ferroptosis can be a potential strategy for TNBC therapy. Next, we explored the average z score of therapeutic drugs (DTP NCI‐60) related to the RNA expression of these ferroptosis regulators, and identified 57 drugs out of 20 348 compounds that showed a significant connection between their IC50 values and the expression levels of these ferroptosis‐related genes (Figure 2C). Interestingly, gambogic acid (GA), a prenylated xanthone moiety isolated from the *Garcinia hanburyi* tree, emerged as a potential inducer of TNBC ferroptotic cell death. As shown in Figure 2C and Figure S3 (Supporting Information), GA exhibited close association with the majority of these ferroptosis‐related genes, and the expression levels of solute carrier family 7 member 11 (SLC7A11), ferritin heavy chain 1 (FTH1), sequestosome1 (SQSTM1), ectonucleotide pyrophosphatase 2 (ENPP2), and caveolin 1 (CAV1) genes were highly correlated with the IC50 value of GA in the TNBC dataset. Previous studies have shown that GA possess potential in inhibiting the growth of various tumors including breast cancers through several mechanisms including apoptosis, autophagy, cell cycle arrest, and the inhibition of invasion.^[^ ^21^ ^]^ Although our previous study also revealed that GA could sharply deplete glutathione (GSH) content within tumor cells, limited research has been conducted on the mechanism by which GA induces ferroptosis in cancer treatment.^[^ ^22^ ^]^ We utilized Swiss Target Prediction and Comparative Toxicogenomics Database to predict potential targets of GA (Table S1, Supporting Information). Notably, thioredoxin (TRX) protein emerged as a promising target for GA, given its close association with several ferroptosis‐related genes (Figure S4, Supporting Information). Among these ferroptosis‐related genes, SLC7A11, FTH1, SQSTM1, ENPP2, and CAV1 exhibited a strong correlation with TRX protein expression (*P* < 0.001, Figure 2B). Thus, GA can be a potential ferroptosis inducer for TNBC. The 3D structure of GA and its molecular docking sites with TRX were depicted in Figure 2F and Table S2 (Supporting Information). Glutamic acid 99 of TRX can form a noncovalent bond with GA. Surface plasmon resonance (SPR) analysis confirmed the affinity of GA to TRX with the affinity constant of 21.8 µm (Figure S5, Supporting Information). MCL1 (Myeloid cell leukemia 1), another potential target of GA (Figure S6, Supporting Information), which can protect cancer cells especially TNBC from apoptosis and decrease their sensitivity to anticancer therapies.^[^ ^23^ ^]^ Similar to TRX protein, MCL1 showed tight associations with numerous ferroptosis‐related genes (Figure S4, Supporting Information). In addition, GO and KEGG enrichment analysis was performed on GA‐treated 4T1 breast cancer cells to investigate the metabolic regulation of GA on TNBC. It was found that ferroptosis‐related pathways including ferroptosis, glutathione metabolism, biosynthesis of unsaturated fatty acids, and mineral absorption showed high enrichment scores (Figure 2D; Figure S7, Supporting Information). Furthermore, we validated that treatment with GA led to a significant reduction in TRX and MCL1 expressions in 4T1 breast cancer cells, resulting in highly efficient killing effect (IC50 = 2.20 µg mL^−1^) against 4T1 cells (Figure S8A,B, Supporting Information). In addition, GA could significantly produce reactive oxygen species (ROS), deplete GSH and ferrous irons, and induce lipid peroxidation in 4T1 cells (Figure S9A–G, Supporting Information). Moreover, ferroptosis inhibitors such as ferrostatin‐1 (Fer‐1), deferoxamine (DFO), and vitamin E could significantly abolish the cytotoxicity induced by GA. We investigated morphological changes in mitochondria using transmission electron microscopy (TEM). The PBS group exhibited a normal mitochondrial morphology, whereas the GA group displayed smaller mitochondria with a decreased number of cristae and increased membrane density (Figure S10, Supporting Information). These alterations are indicative of ferroptotic cell characteristics. These results indicate that GA can be regarded as an effective ferroptosis inducer to enhance the ferroptosis‐immunotherapy of TNBC.

### 2.2 Preparation and Characteristics of Nanomotors

**Figure 3:**
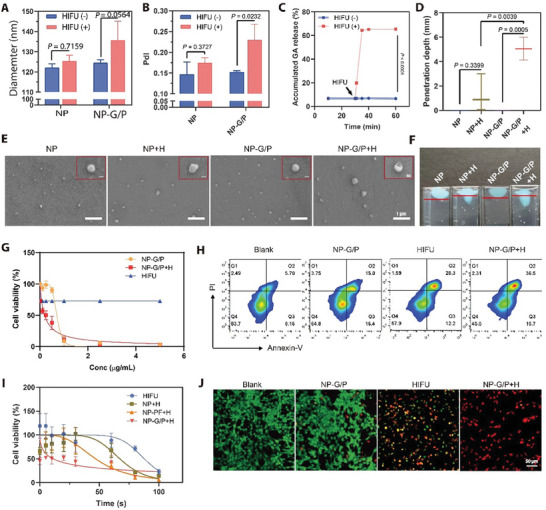
A,B) The size (A) and polydisperse index (PdI) (B) of NP‐G/P under HIFU irradiation (*n* = 3). C) On‐demand GA release from NP‐G/P induced by HIFU treatment (*n* = 3). D) The penetration depth of NP‐G/P after HIFU irradiation in the stimulated tumor extracellular matrix (*n* = 3). E) Scanning electron microscope (SEM) confirmed the morphological change of HIFU‐treated NP‐G/P. The red box referred specifically to changes in the structure of individual particles. F) The propulsion trajectory of NP‐G/P and NP was measured in the stimulated tumor extracellular matrix. G) The effect of HIFU on in vitro cytotoxicity of NP‐G/P was evaluated by the CCK‐8 method (*n* = 3). H) Flow cytometry analysis of tumor cell apoptosis after different treatments. I) The effect of nanomotors on the working efficiency of HIFU therapy was evaluated by the CCK‐8 method (*n* = 3). J) Representative fluorescence images of live (green) and dead (red) 4T1 cells after different treatments. In all cases, significance was defined as *P* ≤ 0.05. The significance of the difference between two groups was determined via Student's *t*‐test. The significance of the difference of more than two groups was determined via One‐way ANOVA test.

The nanomotors (NP‐G/P) were prepared by a nano‐precipitation method. In these nanorobots, GA and PFOB were co‐encapsulated into polymeric PLGA cores which were decorated by DSPE‐PEG2000 to introduce a long circulation effect (NP‐G/P). Scanning electron microscope (SEM) imaging demonstrated that NP‐G/P was well‐dispersed with a spherical structure (**Figure** **3**E). The average hydrodynamic diameter of NP‐G/P was ≈130.8 nm, which was 5.5 nm larger than that of NP and NP‐PF (Figure S11A–D, Supporting Information). Meanwhile, PFOB loading increased the zeta potential of NP‐PF and NP‐G/P to −35 mV and −33 mV compared with that of NP (−42 mV), which might be due to that PFOB presented on the surface of spherical structure partially shields the negative charge of PLGA nanoparticles(Figure S11E, Supporting Information).^[^ ^24^ ^]^ There was no obvious change in the particle size during storage at 4°C within 7 days, suggesting that NP‐G/P possesses a good storage stability (Figure S11B, Supporting Information). The drug loading capability (DLC) and the encapsulation efficiency (EE) of GA in NP‐G/P were 0.79 ± 0.01% and 66.03 ± 0.07%, respectively. The DLC and EE of PFOB in NP‐G/P were 17.37 ± 1.40% and 90.00 ± 2.07%, respectively. As PFOB can confer NP‐G/P responsiveness to HIFU for motion and on‐demand drug release, to illustrate the driving and blasting performance of HIFU‐triggered nanomotors, changes in the physicochemical properties of NP or NP‐G/P with HIFU treatment were evaluated. As shown in Figure 3A,B, the size of NP‐G/P under 8.5 W HIFU treatment for 5 min enlarged with the polydispersity index (PdI) significantly increased from 0.152 to 0.224. However, the HIFU treatment displayed no significant influences on the size distribution of NP. Under SEM, compared with the spherical structure of NP and NP‐G/P themselves, slight structural deformation of NP appeared after HIFU treatment, but NP‐G/P showed obvious morphological disintegration that could contribute to release the cargoes in NP‐G/P (Figure 3E). These results indicate that the PFOB in NP‐G/P has a high cavitation effect under HIFU treatment. Afterward, the release of GA from NP‐G/P in PBS (containing 0.5% Tween‐80) was evaluated. Only 6.65% of GA released from NP‐G/P without any intervention after 1 h but 65.33% of GA released after 5‐min HIFU treatment (8.5 W) (Figure 3C), demonstrating an on‐demand release of GA from NP‐G/P under HIFU irradiation. To investigate the tumor penetration ability of nanomotors driven by HIFU in vitro, fibrin gel was employed as the tumor extracellular matrix and DiD‐labeled NP‐G/P (blue) was added to the surface of matrix to visualize the moving distance repulsed by HIFU. Significant movement was observed immediately under HIFU in the NP‐G/P group compared with other groups (Figure 3F), and the penetration distance of NP‐G/P+H (7.06 mm) was 2.13‐fold longer than that of NP+H (3.31 mm) (Figure 3D). In contrast, HIFU‐untreated groups displayed no noticeable penetration even after 24 h of incubation. Altogether, NP‐G/P combined with HIFU irradiation demonstrated superb penetration properties, nanostructural damage, and rapid drug release, indicating that HIFU triggers active propulsion and on‐demand drug release on NP‐G/P nanomotors.

### 2.3 HIFU Enhanced Cytotoxicity of Nanomotors on Tumor Cells

The cellular uptake of nanomotors was assessed using 4T1 murine breast carcinoma cells as a model for TNBC. Flow cytometry and fluorescence imaging demonstrated that NP‐G/P exhibited time‐dependent cellular uptake, with maximal endocytosis observed after 12 hours of incubation (Figure S12A,B, Supporting Information). The in vitro cytotoxicity of NP‐G/P combined with HIFU treatment was evaluated by the Cell‐Counting Kit 8 (CCK‐8) method. We initially explored appropriate HIFU irradiation parameters by treating 4T1 cells with different powers and times. The cell killing effects of 8.5 W and 10.1 W HIFU were similar when the irradiation time ranged from 10 s to 37 s (Figure S13, Supporting Information). Considering that lower power would result in less damage to culture wells or surrounding normal tissues in vivo, the HIFU parameters of 8.5 W for 30 s were used in the subsequent cell studies. As shown in Figure 3G, the cell viability gradually decreased with increasing GA concentration in all groups except for the HIFU group, while HIFU treatment significantly enhanced the cytotoxicity of nanomotors. The IC50 value of GA for NP‐G/P was 0.74 µg mL^−1^, while that for NP‐G/P+H was reduced to 0.19 µg mL^−1^, indicating that HIFU irradiation exhibits superior effects on improving killing ability of nanomotors to tumor cells. Similarly, it was also found that nanomotors could shorten the HIFU irradiation time and improved the efficiency of HIFU therapy. The IC50 values for NP‐G/P+H, HIFU alone, and NP‐PF+H were 11.09 s, 87.79 s, and 46.82 s respectively (Figure 3I). The highly efficient cell‐killing effect of NP‐G/P+H was further confirmed by a cell apoptosis assay based on Annexin V‐FITC/PI staining. It was revealed that NP‐G/P+H showed the highest percentage of total apoptotic cells (40.57%) compared to other groups (Figure 3H; Figure S14A, Supporting Information). Furthermore, laser scanning confocal microscopy directly visualized the cell‐killing effect after various treatments by staining live cells with calcein‐AM (green) and dead cells with propidium iodide (red). It was found a significant number of 4T1 cells were dead after NP‐G/P+H treatment (Figure 3J; Figure S14B, Supporting Information), which was well consistent with the above flow cytometry results. All these results indicate that these HIFU‐driven nanomotors possess superb antitumor effects in vitro.

### 2.4 HIFU Drove Nanomotors to Trigger Effective Ferroptosis of Tumor Cells

**Figure 4:**
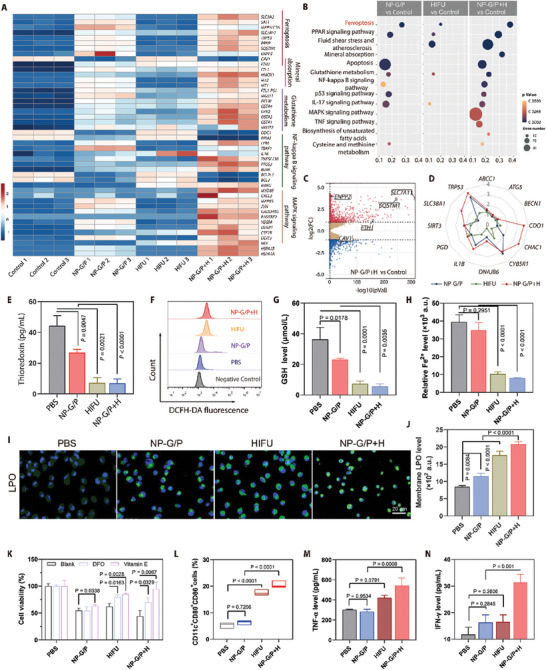
A) The heat map of typical differentially expressed genes in 4T1 cells after different treatments through the RNA‐sequencing analysis (*n* = 3). The number in the scale plate referred to the |log_2_fold change|. B) The top 13 pathways in which differentially expressed genes in 4T1 cells were enriched by the KEGG enrichment analysis. C) The volcano map showed the identified upregulated and downregulated genes induced by NP‐G/P treatment compared with PBS treatment (*n* = 3). The screening criteria were FDR < 0.05 and |log_2_fold change|> = 1. D) The radar chart of typical differentially expressed ferroptosis‐driver genes in 4T1 cells after different treatments through the RNA‐sequencing analysis (*n* = 3). The number in the radar chart referred to the |log_2_fold change|. E) The intracellular thioredoxin expression in 4T1 cells after different treatments (*n* = 3). F) Relative ROS levels in 4T1 cells after various treatments after staining with DCFH‐DA. G) Quantitative analysis of intracellular GSH concentration in 4T1 cells after various treatments (*n* = 3). H) The relative expression level of ferric (II) in 4T1 cells after various treatments (*n* = 3). I) Fluorescence imaging of 4T1 cells with various treatments after staining with BODIPY‐C11, showing the intracellular distribution of LPO (green fluorescence). J) The intracellular LPO level in 4T1 cells after various treatments was measured by flow cytometry (*n* = 3). K) The cell viability of 4T1 cells that received different treatments in the presence of different ferroptosis inhibitors including DFO and vitamin E (*n* = 3). L) Flow cytometry analysis of CD80^+^CD86^+^ BMDC cells in different groups (*n* = 3). M,N) The expression levels of TNF‐α (M) and IFN‐γ (N) secreted from BMDCs co‐incubating with 4T1 cells after various treatments (*n* = 3). In all cases, significance was defined as *P* ≤ 0.05. The significance of the difference between two groups was determined via Student's *t*‐test. The significance of the difference of more than two groups was determined via One‐way ANOVA test.

Previous studies have shown that HIFU can kill tumor cells by heat effect and disruption of cellular metabolism (eg. alteration of cellular redox status).^[^ ^25^ ^]^ However, the specific mode of cell death caused by HIFU and its ability to drive ferroptosis in tumor cells remain unknown. To elucidate the mechanisms underlying the enhanced cytotoxicity of nanomotors on tumor cells with HIFU treatment, we used RNA‐sequencing to determine gene expression changes in tumor cells after NP‐G/P+H treatment (**Figure** **4**A–D). Pathway enrichment analysis revealed that compared to the control group (PBS treatment), HIFU treatment significantly upregulated pathways related to ferroptosis, fluid shear stress and atherosclerosis, glutathione metabolism, PPAR signaling pathway, and IL‐17 signaling pathway in tumor cells. Furthermore, compared to the NP‐G/P group, the NP‐G/P+H group showed significant upregulation of pathways related to ferroptosis, fluid shear stress and atherosclerosis, mineral absorption, glutathione metabolism, and inflammatory signaling pathway (Figure 4B; Figure S15A,B, Supporting Information), indicating that HIFU not only induces cell ferroptosis and activates immune response, but also drives the cell ferroptosis effect of GA. Heat map analysis showed 24 genes related to ferroptosis changed significantly when treated with NP‐G/P; among them 9 genes were upregulated in glutathione metabolism while 6 genes were upregulated in mineral absorption by KEGG analysis (Figure 4A). Especially, compared with control, HIFU treatment significantly increased expressions of ferroptosis‐suppressor genes SLC7A11, SQSTM1, and FTH1 which are sensitive genes for GA by 6.47‐fold, 2.34‐fold, and 2.28‐fold, respectively (Figure 4A,C). More importantly, the expressions of SLC7A11 and SQSTM1 were significantly increased in the NP‐G/P+H group compared with NP‐G/P and HIFU groups (Figure 4A). In addition, NP‐G/P+H treatment could significantly upregulate the expressions of a series of ferroptosis‐driver genes including DNAJB6 (DnaJ heat shock protein family Hsp40 member B6), IL1B (interleukin 1 beta), PGD (phosphogluconate dehydrogenase), TRP53 (transformation related protein 53), CHAC1 (cation transport regulator 1), SLC38A1 (microtubule‐associated protein 1 light chain 3 alpha), and CDO1 (cysteine dioxygenase type 1) compared with NP‐G/P treatment (Figure 4D). These results indicate HIFU not only sensitizes tumor cells to GA through upregulating three ferroptosis‐suppressor genes, but also drives tumor cell ferroptosis through upregulating many ferroptosis‐driver genes.

Afterward, ferroptosis of tumor cells after NP‐G/P+H treatment was verified in vitro. Considering that thioredoxin is the main target of GA and thioredoxin is tightly negatively associated with intracellular ROS level and cell ferroptosis (Figure 2B), the intracellular thioredoxin level in tumor cells after NP‐G/P+H treatment was first determined.^[^ ^26^ ^]^ The results revealed that NP‐G/P treatment could significantly deplete thioredoxin in 4T1 cells by 39.3% while HIFU and NP‐G/P+H treatments sharply deplete thioredoxin in cells by 83.7% and 84.4%, respectively compared to PBS treatment (Figure 4E). Moreover, we explored the expressions of several ferroptosis‐related proteins, including ACSL4 (acyl‐CoA synthetase long‐chain family member 4), PTGS2 (prostaglandin‐endoperoxide synthase 2), and GPX4. It was found that either GA or NP‐G/P had no significant effect on the expressions of these three proteins, while HIFU and NP‐G/P+H were able to significantly downregulate their expressions (Figures S9H–K and S16, Supporting Information). Then, the concentrations of ferroptosis‐related markers including intracellular oxidative stress‐associated markers ROS and GSH, iron metabolism marker Fe^2+^, and the key ferroptosis marker LPO (lipid peroxide) were quantitatively evaluated. It was found that NP‐G/P+H treatment significantly increased the ROS level in tumor cells by 9.1 folds and 2.1 folds compared with PBS and NP‐G/P treatments, respectively (Figure 4F; Figure S17, Supporting Information). The HIFU treatment also produced high levels of intracellular ROS, which were lower than those in the NP‐G/P+H group. Meanwhile, in contrast to PBS treatment, NP‐G/P, HIFU, and NP‐G/P+H treatments significantly depleted the intracellular GSH level by 23.1%, 75.7%, and 84.6%, respectively (Figure 4G), indicating that HIFU strengthens the GSH depletion effect of NP‐G/P. These results suggest that the NP‐G/P treatment can disturb the intracellular redox equilibrium and that the HIFU can amplify its effect. The sharply reduced ferrous ion level in the NP‐G/P+H group once again proved that there was excessive ROS production in 4T1 cells which could oxidize ferrous ion to ferric ion (Figure 4H). NP‐G/P+H treatment leads to a surge in intracellular oxidative stress and may enhance the lipid peroxidation on biomembranes. It was found that NP‐G/P+H treatment displayed the highest LPO level determined by flow cytometry and fluorescence imaging (Figure 4I,J). TEM analysis demonstrated that treatment of 4T1 cells with either NP‐G/P or HIFU resulted in significant damage to the mitochondria. This damage included characteristics such as mitochondrial shrinkage, a decrease in the quantity of cristae, and an increase in membrane density, when compared to the control group (Figure S10, Supporting Information). Furthermore, when 4T1 cells were treated with the combination of NP‐G/P and HIFU (NP‐G/P+H), the detrimental effects on the mitochondria were further heightened. These findings align with the typical features associated with cell ferroptosis. Finally, tumor cell ferroptosis treated with NP‐G/P+H was validated by ferroptosis inhibitors. As shown in Figure 4K and Figure S18 (Supporting Information), the addition of common ferroptosis inhibitors such as DFO, vitamin‐E, and Fer‐1 significantly abolished NP‐G/P+H‐induced cytotoxicity of 4T1 cells. All these results indicate that the tumor cells after NP‐G/P+H treatment undergo ferroptosis.

Next, the immune effects triggered by tumor ferroptosis were investigated in vitro. It is known that ferroptotic cells can stimulate antitumor immunity by releasing damage‐associated molecular patterns (DAMPs). This includes the secretion of adenosine triphosphate (ATP), the release of high mobility group box‐1 (HMGB‐1), and the translocation of calreticulin (CRT), all of which contribute to the maturation of dendritic cells (DCs).^[^ ^27^ ^]^ As illustrated in Figure S19 (Supporting Information), both NP‐G/P and GA induced a certain level of ATP release, with HIFU demonstrating a stronger effect. The combined treatment groups, GA+H and NP‐G/P+H, exhibited the most potent impact. Similarly, the release of HMGB‐1 and the expression level of CRT on the cell membrane were most significant in the NP‐G/P+H and GA+H groups among all treatments. Immature DCs extracted from mouse bone marrow cells were co‐cultured with 4T1 cells after different treatments and the CD80/CD86 expression on the DCs was assessed. The NP‐G/P+H‐treated tumor cells significantly enhanced the surface expression of maturation markers on DCs (CD80^+^CD86^+^) by 3.86, 3.27, and 1.17 folds compared with PBS, NP‐G/P, and HIFU‐treated tumor cells, respectively (Figure 4L; Figure S20, Supporting Information), indicating HIFU can promote tumor cell ferroptosis‐triggered DC maturation. Importantly, NP‐G/P+H treatment increased TNF‐*α* secretion by bone marrow‐derived dendritic cells (BMDCs) by 1.80, 1.91, and 1.29 folds compared with PBS, NP‐G/P and HIFU treatments, respectively (Figure 4M). Similar responses in IFN‐γ secretion by BMDCs were also observed (Figure 4N). The above data therefore provide evidence that 4T1 cells treated with NP‐G/P+H are more effective in stimulating DC maturation.

### 2.5 Improved Tumor Accumulation and Penetration of NP‐G/P Driven by HIFU in TNBC Models

The pharmacokinetics behavior of NP‐G/P was investigated. It was shown that NP‐G/P had a pharmacokinetics profile similar to NP (Figure S21 and Table S3, Supporting Information). There were no significant differences in the pharmacokinetics parameters between NP‐G/P and NP, indicating that the loading of GA and PFOB in nanoparticles does not significantly influence their pharmacokinetics behavior.

It has been shown that HIFU can induce active propulsion and blasting capability on NP‐G/P nanomotors in vitro. To verify this effect at the organ level, we injected nanomotors into the pork liver and subjected it to HIFU irradiation. The ablation volume of the pork liver in the NP‐G/P+H group (282.4 mm^3^) was significantly larger than that of the PBS (74.9 mm^3^) and NP (97.4 mm^3^) groups (Figure S22A,B, Supporting Information), primarily due to rapid vaporization and vibration of PFOB in nanomotors triggered by HIFU irradiation.

**Figure 5:**
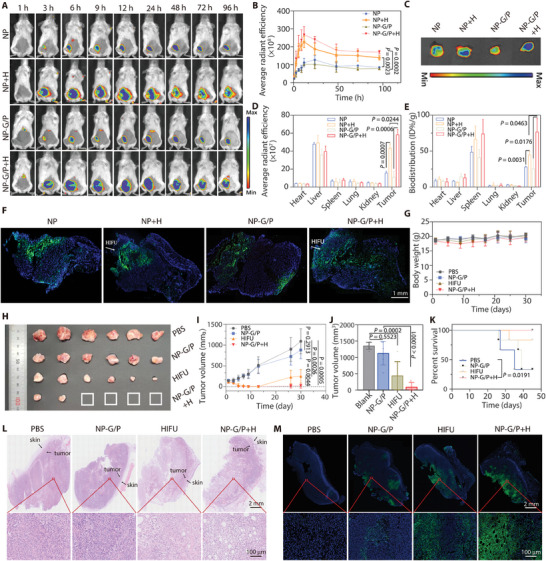
A) Representative in vivo fluorescence images of 4T1 tumor‐bearing mice after intravenous injection of NP or NP‐G/P at different time points, and HIFU treatment was performed at 3 h after NP or NP‐G/P injection. B) Fluorescence signals at tumor regions were plotted with time based on in vivo fluorescence imaging (*n* = 3). C) Ex vivo imaging of major organs and tumors at 24 h after injection. D) Semi‐quantitative biodistribution of nanomotors in major organs and tumors at 24 h after injection obtained from ex vivo imaging (*n* = 3). E) Quantitative biodistribution of nanomotors in major organs and tumors at 24 h after injection (*n* = 3). F) Representative immunofluorescence images of tumor slices at 12 h after injection to reveal the accumulation and penetration pattern of nanomotors in tumors. Blue: cell nuclei; Green: nanoparticles. G) Changes of the body weight of mice after various treatments within 30 days (*n* = 6). I) Tumor growth curves of mice after different treatments within 30 days (*n* = 6). H,J) Photograph images H) and the tumor volume J) of excised tumors at 30 days in various treatment groups (*n* = 6). K) Survival curve of 4T1 tumor‐bearing mice within 45 days (*n* = 6). L,M) Representative histochemical images (H&E staining) L) and cell apoptosis images (TUNEL staining) (M) of tumor slices obtained at the 7th day after various treatments. Red boxes in the upper row were shown in the lower row with high magnification. In all cases, significance was defined as *P* ≤ 0.05. The significance of the difference between two groups was determined via Student's *t*‐test. The significance of the difference of more than two groups was determined via One‐way ANOVA test.

Efficient HIFU‐driven penetration of nanomotors into tissues enables them to accumulate inside tumors efficiently, enhancing therapeutic drug efficacy against tumors. We investigated accumulation and penetration of DiD‐labeled NP‐G/P in 4T1 tumor‐bearing mice after HIFU irradiation; fluorescence imaging revealed a significant increase in fluorescence intensity at tumor sites for both NP and NP‐G/P over time post‐injection with highest values observed at 12 h for all groups receiving HIFU treatment (**Figure** **5**A,B). It could be found that the fluorescence signal at the tumor site was gradually enhanced with time and reached the highest value 12 h post‐injection for the NP‐G/P, NP+H, and NP‐G/P+H groups (Figure 5A,B). Notably, fluorescence intensity was significantly stronger by two‐ or three‐fold for the NP‐G/P+H or NP+H groups compared to the control groups, as evidenced by AUC_0‐t_ values at tumor sites (Figure S23, Supporting Information). *Ex vivo* imaging also demonstrated that the accumulation of nanoparticles in tumors for the NP‐G/P+H group was significantly higher than that for any other group (Figure 5C,D; Figure S24, Supporting Information). Further quantification of biodistribution revealed that NP or NP‐G/P showed a relatively weak accumulation in tumors while HIFU irradiation significantly increased NP or NP‐G/P accumulation in tumors by 1.61 or 3.00 folds (Figure 5E). Notably, the NP‐G/P+H group had significantly higher nanoparticle accumulation in the tumor than the NP+H group. Subsequently, the penetration pattern of nanomotors in tumors driven by HIFU was directly visualized in frozen sections (Figure 5F). It could be observed that most of the nanoparticles were located at the edge of the tumor edge in the NP, NP+H, or NP‐G/P groups. In contrast, the nanoparticles in the NP‐G/P group were located along the HIFU irradiation direction and penetrated across the tumor. These results indicate that HIFU irradiation not only greatly enhances nanomotor accumulation in tumors, but also highly promotes nanomotor penetration in tumors, possibly due to that HIFU irradiation increasing tumor vessel permeability and driving nanomotor penetration ability.

### 2.6 Improved Antitumor Efficacy of NP‐G/P Driven by HIFU on TNBC Models

The antitumor experiments were subsequently investigated in 4T1 tumor‐bearing mouse models (Figure S25, Supporting Information). As shown in Figure 5H–J and Figures S26 and S27 (Supporting Information), tumor growth was significantly delayed after NP‐G/P treatment. HIFU treatment exhibited excellent ability against solid tumors within 10 days, presumably through physical destruction, but the tumor relapsed on day 13 due to incomplete ablation. Notably, NP‐G/P+H treatment showed a significantly stronger inhibitory effect on tumor growth than NP‐G/P or HIFU therapy, with 66.7% of tumors showing complete regression. Survival monitoring demonstrated that all mice in the NP‐G/P+H group survived for 45 days, compared to 33.3% and 83.3% in the NP‐G/P and HIFU groups, respectively (Figure 5K). In all four groups, the body weight of mice in different groups remained similar throughout the course of the treatment (Figure 5G), suggesting that no obvious adverse effects result from NP‐G/P+H treatment. The therapeutic effect of different treatments on tumor tissue was evaluated in depth using Hematoxylin and Eosin (H&E) staining and TUNEL assay. As shown in Figure 5L,M, varying degrees of cell death were observed in the PBS, NP‐G/P, HIFU, and NP‐G/P+H groups. Negligible necrosis appeared in the PBS and NP‐G/P groups. The much greater necrosis with pyknosis and karyorrhexis in the NP‐G/P+H group can be mainly attributed to the synergistic killing effect of GA and HIFU on tumor cells. TUNEL‐positive cells in the different treatment groups were consistent with the H&E staining results, with the NP‐G/P+H group showing the broadest apoptosis signal at the interface of the tumor disruption. These results suggest that HIFU enhances the antitumor efficacy of NP‐G/P.

### 2.7 HIFU Drove NP‐G/P to Activate Ferroptosis‐Mediated Antitumor Immunity in Metastatic TNBC Models

**Figure 6:**
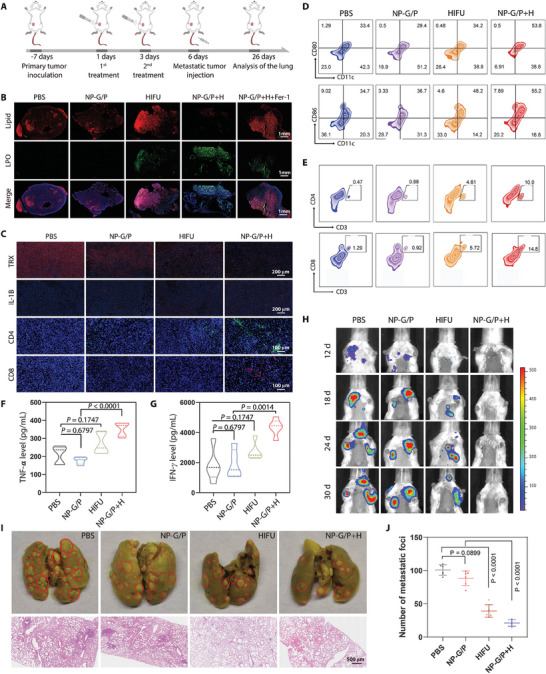
A) Schematic illustration of the lung metastasis establishment and therapeutic regimen. B) Representative immunofluorescence images of tumor slices stained with C11‐BODIPY after different treatments. Blue: cell nuclei; Green: LPO; Red: normal lipid. C) Representative immunofluorescence images of CD4^+^ T cells, CD8^+^ T cells, TRX, and IL‐1 in primary tumors. Blue: cell nuclei; Green: CD4^+^ T cells; Red: CD8^+^ T cells, TRX, and IL‐1. D) Representative flow cytometry charts of CD80^+^ and CD86^+^ in CD11c^+^ DCs from the tumor‐draining lymph nodes after different treatments. E) Representative flow cytometry charts of CD4^+^ and CD8^+^ T cells in primary tumors after different treatments. F,G) Concentrations of TNF‐α F) and IFN‐γ G) in tumors from various treatment groups (*n* = 5). H) Bioluminescence imaging of mice showing the growth of lung metastases in mice after various treatments. I) Representative photographs of lungs after staining with Bouin's solution and representative histochemical images of lung tissue slices from mice in various treatment groups. J) The pulmonary metastasis nodule numbers in the lung from mice in various treatment groups (*n* = 6). In all cases, significance was defined as *P* ≤ 0.05. The significance of the difference of more than two groups was determined via One‐way ANOVA test.

Encouraged by the efficient tumor delivery and strong tumor eradication efficacy of these HIFU‐driven nanomotors, we next investigated whether they could activate ferroptosis‐mediated antitumor immunity in metastatic TNBC models (**Figure** **6**). The treatment regimen is shown in Figure 6A. As shown in Figure 6B, NP‐G/P or HIFU treatment resulted in faint or weak LPO signals only at the edge of the tumor and in a large region of normal lipid. In comparison, NP‐G/P+H treatment significantly increased the amount of LPO in tumor tissue, but decreased the amount of normal lipids. The addition of Fer‐1 restored LPO and normal lipid levels to levels similar to those achieved with HIFU monotherapy by cutting off the glutathione pathway, suggesting that effective ferroptosis occurs in the inner tumor region with the assistance of HIFU‐driven nanomotors.

Consistent with the in vitro result, it was observed that NP‐G/P treatment exhibited slight inhibition on the TRX expression in tumors due to the targeted binding of GA with TRX, and HIFU and NP‐G/P+H treatments dramatically decreased the TRX expression in tumors compared with PBS treatment (Figure 6C). These results suggest that HIFU enhances the perturbative effect of NP‐G/P on the intracellular redox equilibrium though inhibiting TRX. The in vitro transcriptomics analysis revealed that HIFU irradiation enriched some differentially expressed genes in the NF‐kappa B pathway (Figure 4A), which cooperatively induces the expression of IL1‐type cytokines and promotes the antitumor activity of both CD4 and CD8 T cells.^[^ ^28^ ^]^ Interestingly, it was found that HIFU and NP‐G/P+H treatments significantly increased IL1 expression in tumors compared with PBS treatment (Figure 6C), indicating HIFU helps to reshape the tumor immune microenvironment.

It should be noted that cell ferroptosis oftentimes leads to the release of various immunogenic damage‐associated molecular patterns (DAMP) that can enhance immune responses.^[^ ^14^, ^29^ ^]^ We then investigate the immune activation effect of NP‐G/P+H‐triggered ferroptosis. It was found that NP‐G/P+H treatment could significantly promote the maturation of DCs inside the lymph nodes adjacent to the primary tumors (Figure 6D; Figure S28A,B, Supporting Information) compared with other treatments. Additionally, NP‐G/P+H treatment could significantly promote the infiltration of CD3^+^CD4^+^ and CD3^+^CD8^+^ T cells in tumors compared with other treatments (Figure 6C,E; Figure S28C,D, Supporting Information). Moreover, the secretion levels of cytotoxic cytokines including tumor necrosis factor alpha (TNF‐α) and interferon gamma (IFN‐γ) in the tumors after NP‐G/P+H treatment were also significantly increased (Figure 6F,G). Therefore, an adaptive immune response was initiated following NP‐G/P+H therapy by inducing DC maturation and T cell expansion and infiltration into tumors and HIFU irradiation significantly enhanced NP‐G/P‐induced antitumor immunity through reshaping the tumor immune microenvironment. Encouraged by the strong systemic antitumor immunity activated by NP‐G/P+H treatment, we evaluated the effect of this activated immunity on the inhibition of lung metastasis in TNBC. After the primary tumor was treated with NP‐G/P+H, 4T1‐Luc cancer cells were subsequently injected intravenously to mimic the escape of tumor cells from the primary tumor site to the lung. Bioluminescence imaging demonstrated that NP‐G/P+H treatment significantly inhibited tumor growth of lung metastases, while HIFU or NP‐G/P treatment had modest impact on the growth of lung metastases (Figure 6H; Figure S29, Supporting Information). The number of visible tumor nodules on the lung surface in the NP‐G/P+H group was significantly decreased by ≈2.6‐fold and 1.6‐fold as compared to that in the NP‐G/P and HIFU groups, respectively (Figure 6I,J). Indicated by H&E‐stained lung sections, there was the slightest inflammation and necrosis in the lung tissues of the NP‐G/P+H group among all treatment groups (Figure 6I). These results demonstrate that NP‐G/P+H‐triggered ferroptosis can not only effectively inhibit the growth of primary breast tumors, but also could enhance the antitumor immune response to inhibit the growth of tumor metastases. Considering that tumor metastasis is responsible for the breast cancer‐related mortality, HIFU‐driven nanomotors that activate ferroptosis‐mediated antitumor immunity are promising for restraining tumor metastasis and prolonging overall patient survival.

### 2.8 Assessment of the Biosafety

4T1 tumor‐bearing mice treated with various therapies were sacrificed after 20 days and major organs including the heart, liver, spleen, lung, and kidneys were harvested for H&E staining. No obvious pathological abnormalities were observed in the NP‐G/P+H group, whereas mice treated with PBS, NP‐G/P, or HIFU displayed inflammation (Characteristics of tumor cell metastasis) in the lung tissue (Figure S30, Supporting Information). The results of routine blood analyses showed that the lymph ratio in the NP‐G/P+H group was significantly increased to the normal range compared with that in the PBS group (Figure S31A, Supporting Information). Moreover, compared with PBS treatment, NP‐G/P+H treatment resulted in the reduction of WBC, RBC, and HGB to the normal ranges (Figure S31B–D, Supporting Information). Other panels from the blood analysis presented minimal discrepancies in all groups (Figure S31E–H, Supporting Information). Furthermore, we conducted a comprehensive assessment of the toxicological impact of NP‐G/P on healthy BALB/c mice. Blood samples and major organs were collected seven days post two intravenous administrations of NP‐G/P. Blood biochemical analysis demonstrated that the liver and kidney function indices in the NP‐G/P treatment group were maintained within normal ranges, exhibiting no significant deviations when compared to the control group (Figure S32, Supporting Information). The H&E staining of vital organs further revealed an absence of significant damage (Figure S33, Supporting Information). These results all indicate that NP‐G/P+H does not cause significant side effects in mice and is a safe and effective treatment method.

## 3 Discussion

Effective inducers of ferroptosis are powerful tools for treating apoptosis‐resistant cancers, including TNBC. The development of drugs that specifically trigger ferroptosis in TNBC patients holds immense value. In this study, we presented a screening strategy to identify regulators of ferroptosis, which yielded a collection of potential drugs for TNBC treatment. This method has been instrumental in the discovery of potential ferroptosis drugs and elucidation of key ferroptosis regulators and related pathways. In the variety of candidate drugs available to induce ferroptosis, gambogic acid, as a traditional drug, demonstrated great potential in TNBC therapy targeting ferroptotic cell death. Consequently, GA was incorporated into HIFU‐driven nanomotors responsive to HIFU irradiation, resulting in efficient deep penetration and induction of ferroptotic cell death in TNBC cells. It was found that HIFU not only activated nanomotors to effectively induce ferroptotic cell death by sensitizing TNBC cells to GA and upregulating numerous genes associated with driving ferroptosis but also triggered robust antitumor immunity mediated by ferroptotic cell death in primary and metastatic models of TNBC, leading to substantial tumor regression and prevention of metastasis. Thus, the versatility offered by the HIFU‐driven nanomotor platform enables its application for delivering other candidate inducers of ferroptosis toward developing precision treatments for TNBC.

It is important to acknowledge that further exploration is required. Although our study demonstrated that GA induced effective ferroptotic cell death by inhibiting thioredoxin activity and increasing lipid peroxidation levels within TNBC cells, additional research is needed to fully clarify the specific mechanisms underlying GA‐induced ferroptotic cell death. It is noteworthy that GA has other predictive targets and may induce TNBC ferroptosis through different pathways. Moreover, the HIFU‐driven nanomotor platform boosted the ferroptosis‐immunotherapy effect of GA on TNBC by improving drug accumulation and penetration in tumors, as well as activating effective ferroptosis‐mediated antitumor immunity. The design principle can be readily applied to other potential ferroptosis inducers whose activity correlates significantly with the expression levels of typical ferroptosis‐related genes in TNBC. Further investigation is needed to determine whether they can induce more effective ferroptosis or have a stronger synergistic function with HIFU for TNBC's ferroptosis‐immunotherapy.

## 4 Experimental Section

### Materials

Hydroxyl‐terminated poly (lactic‐co‐glycolic acid) (PLGA‐OH; 0.67 dL/g, 50:50 ratio) was from Lactel (USA). Gambogic acid (GA), 2‐(4‐Amidinophenyl)−6‐indolecarbamidine dihydrochloride (DAPI), the Cell Counting Kit‐8 (CCK8), Annexin V‐FITC/PI Cell Apoptosis Kit, Calcein‐AM/PI staining kit, 2,7‐dichlorofuorescin diacetate (DCFH‐DA), perfluorooctyl bromide (PFOB), D‐luciferin potassium salt, and the GSH/GSSG assay kit were purchased from Beyotime Biotechnology (China). 1,2‐distearoyl‐sn‐glycero‐3‐phosphoethanolamine‐N‐[methoxy(polyethylene glycol)−2000] (DSPE‐PEG2000) was from Laysan Bio Co (USA). Perfluoro‐15‐crown‐5‐ether and deuterochloroform (CDCl_3_) were purchased from Sigma (USA). Dulbecco's modified Eagle's medium (DMEM), RPMI‐1640 cell culture medium, granulocyte‐macrophage colony‐stimulating factor (GM‐CSF), interleukin‐4 (IL‐4), fetal bovine serum (FBS), penicillin streptomycin (PS), Trypsin‐EDTA (0.25%), and 1× PBS (7.4) were all purchased from Gibco (USA). Anti‐CD11c‐FITC, anti‐CD80‐PE, anti‐CD86‐APC, anti‐CD3‐PerCP, anti‐CD4‐FITC, and anti‐CD8‐AF647 were purchased from BioLegend (USA). Anti‐IL‐1 beta antibody, anti‐ACSL4 antibody, anti‐PTGS2 antibody, anti‐GPX‐4 antibody, and anti‐thioredoxin antibody were purchased from Abcam (UK). FerroOrange (Fe^2+^ indicator) was from Maokangbio (China). HMGB‐1, TRX, and MCL1 ELISA assay kit were from Jianglai Biotechnology (China). C11‐BODIPY was obtained from Invitrogen (USA). TNF‐α ELISA kit and IFN‐γ ELISA kit were from Multisciences (China). Fibrinogen and thrombin were obtained from Shanghai Yuanye Biotechnology (China). Thioredoxin (TRX) was obtained from MedChemexpress (USA). Anti‐calreticulin recombinant rabbit monoclonal antibody and iFluor 488 conjugated goat anti‐rabbit IgG goat polyclonal antibody were obtained from Huabio (China). Bouin's fixative solution was purchased from SenBeiJia Biological Technology (China). 4% paraformaldehyde was from Biosharp (China).

### Animals and Cells

BALB/c female mice aged 4–6 weeks were purchased from the Shanghai SLAC Laboratory Animal Ltd (China). All mice were hold in a housing room with temperature of around 22°C and the light cycle from 08:00 to 20:00, and were maintained on a standard diet and water. All animal experiments were performed according to the protocol (2017‐03‐YJ‐PZQ‐01) approved by the Ethics Committee of Fudan University.

Mouse mammary breast carcinoma cells, 4T1 cell lines and 4T1‐luc cell lines were purchased from American Type Culture Collection (ATCC, USA). Both cells were cultured in DMEM supplemented with 10% fetal bovine serum, 1% penicillin/streptomycin at 37°C humidified condition with 5% CO_2_.

### Identification of Ferroptosis Regulators Associated with TNBC

We identified 47 typical ferroptosis‐associated genes from the ferroptosis pathway map in the Kyoto Encyclopedia of Genes and Genomes (KEGG, http://www.genome.jp/kegg/pathway.html) database and divided them into two groups based on their function: ferroptosis drivers or suppressors referring to the FerrDb database (http://www.zhounan.org/ferrdb/current/). Gene expressions of these genes on TNBC and normal breast tissues were obtained from Fudan University Shanghai Cancer Center TNBC datasets (FUSCCTNBC, http://www.biosino.org/; OEP000155 node; GEO: GSE118527; SRA: SRP157974; Figureshare: https://doi.org/10.6084/m9.Figureshare.19783498.v5). Gene Ontology (GO) was performed using the Metascape database (http://metascape.org/). A protein‐protein interaction (PPI) network was constructed based on relationship data obtained from STRING database (https://cn.string‐db.org/) and further processed with R packages “ggraph” and “igraph”.

### Exploration and Validation of Potential Ferroptosis Inducers

The data of 47 predicted ferroptosis drug targets and their RNA composite expression were obtained from the CellMiner database (https://discover.nci.nih.gov/cellminer/home.do). The average z score of compound activity (DTP NCI‐60) was used to conduct the correlation analysis with the RNA composite expression of the ferroptosis regulators.

Target genes of gambogic acid (GA) were analyzed by using the SwissTargetPrediction database (http://www. swisstargetprediction.ch/) and the Comparative Toxicogenomics Database (CTD, https://ctdbase.org/), both of which can predict protein targets of small molecules, to find targets identified with data from both sources. CB‐Dock was used to conduct semiflexible docking to study the interaction between gambogic acid and target proteins such as TRX and MCL1 (https://cadd.labshare.cn/cb‐dock2/php/show_auto_blinddock.php?user=guest&id=87158db11f9aaf75c227c32915bb77fc&token=1697586777127). The structure of gambogic acid was downloaded from the PubChem database with transcoding using Open Babel, and the 3D structure information of the TRX and MCL1 proteins was acquired from the protein database PDB.

To validate whether GA could induce TNBC ferroptosis, 4T1 cells as model TNBC cells were seeded onto a 6‐well plate, cultured overnight, and then subjected to GA (0.5 µg mL^−1^) or PBS treatment for 12 h. Cells were collected for RNA extraction using Trizol reagent following the manufacturer's procedure. Afterward, mRNA was purified from total RNA using Dynabeads Oligo (dT) (Thermo Fisher, CA, USA) with two rounds of purification, reverse‐transcribed to cDNA, and next used to synthesize U‐labeled second‐stranded DNAs. After the heat‐labile UDG enzyme (NEB, cat.m0280, USA) treatment, the ligated products were amplified with PCR. At last, the 2 × 150 bp paired‐end sequencing (PE150) was performed on an Illumina NovaseqTM 6000 (LC‐Bio Technology CO., Ltd., Hangzhou, China) by the vendor's recommended protocol. The differentially expressed genes that were significantly enriched in GO terms and metabolic pathway were performed by GO functional enrichment and KEGG analysis.

### Surface Plasmon Resonance (SPR) Analysis

The interactions between GA and TRX were examined by SPR (OpenSPRTM, Nicoya, Canada). Specifically, the sensor chip was first loaded and the TRX sample was injected according to the instructions. A gradient of GA concentrations was injected to pass over the sensor chip at a constant flow rate of 20 µL min^−1^ in the running buffer composed of 1%DMSO in PBST (pH 7.4). All the experiment was executed at 25 °C. The TraceDrawer (Ridgeview Instruments AB, Sweden) was used to analysis the experimental data under the one‐to‐one analysis model.

### Western Blotting Analysis

Proteins were extracted using Lysis and Extraction Buffer. Protein samples were collected from the supernatants and boiled for 10 min. Equal amounts of each sample were applied to 10% SDS‐polyacrylamide gels for electrophoretic separation and then were transferred to PVDF membranes. The membrane was blocked for 1 h, incubated overnight at 4 °C with the primary antibody (Anti‐ACSL4, anti‐PTGS2, and anti‐GPX4), and then incubated for 2 h with the secondary antibody. The anti‐GAPDH antibody was used as a loading control. The bands were revealed by X‐ray film and analyzed with the Gel‐Pro Analyzer for densitometry analysis.

### Transmission Electron Microscopy (TEM)

4T1 cells were seeded onto a 6‐well plate, cultured overnight, and then subjected to GA/NP‐G/P (0.5 µg mL^−1^) or PBS treatment for 8 h. Then, the GA+H and NP‐G/P+H groups received 8.5 W HIFU treatment for 30 s and were cultured for another 12 h. The tumor cells were collected and fixed with 2.5% glutaraldehyde, the results were taken using TEM (Hitachi HT7800, Japan).

### Preparation and Characterization of Nanomotors

Nanomotors were prepared through a nanoprecipitation method. Briefly, DSPE‐PEG 2000 (1 mg), PLGA‐OH (10 mg), PFOB (1.93 mg), and GA (0.12 mg) were dissolved in 1 mL of acetone as the organic phase. Afterward, the organic phase was injected rapidly to 2 mL of deionized water. After removing acetone under vacuum, nanomotors (NP‐G/P) were obtained. Nanoparticles without PFOB and GA (NP) were prepared as described above except that PFOB and GA were not added to the organic phase. DiD‐labeled NP, NP‐G/P were prepared with the same procedure except that 10 µg of DiD was added to the organic phase in advance.

The hydrodynamic diameter and zeta potential of NP‐G/P were measured by dynamic light scattering (DLS) using a Zetasizer Nano‐ZS analyzer (Malvern, England). The stability of NP‐G/P in vitro was assessed by monitoring the particle size for 1 week at 4°C in phosphate‐buffered saline (PBS). The surface morphology of NP‐G/P and the corresponding changes after HIFU irradiation (CZF‐200, Chongqing Haifu Technology, China) at 8.5 W for 5 min was observed under a scanning electron microscope (SEM) (ZEISS Gemini 300, Germany). The changes in diameter and polydispersity index (PdI) of NP‐G/P after HIFU irradiation were also determined by DLS. Drug loading and encapsulation efficiency of GA in NP‐G/P were detected by ultraviolet (UV) spectrophotometry. In brief, NP‐G/P was spin down under centrifugation (21 000 *g*) for 20 min, dissolved in methanol, and then spin down under centrifugation (21 000 *g*) for 10 min. The supernatant was detected at 360 nm with a UV spectrophotometer (PerkinElmer, USA). The amount of PFOB in the NP‐G/P was determined by ^19^F NMR by dissolving NP‐G/P into CDCL_3_ containing perfluoro‐15‐crown‐5‐ether as an internal standard to estimate the drug loading and encapsulation efficiency of PFOB in NP‐G/P as previously described.^[^ ^30^ ^]^ To demonstrate the HIFU‐responsive GA release, NP‐G/P was suspended in PBS with 0.5% Tween‐80 and received HIFU treatment (8.5 W for 5 min), and the accumulated amount of GA released from NP‐G/P was detected by UV spectrophotometry at different time points.

### In Vitro Penetration Ability of Nanomotors under HIFU Irradiation

To verify the motor penetration effect under HIFU irradiation, a soft fibrin gel was formed to simulate the tumor matrix after mixing fibrinogen (2 mg mL^−1^) with thrombin (10 U mL^−1^) for 90 s at 37 °C in glass tubes. After loading NP‐G/P in the well of the gel, HIFU irradiation (8.5 W, 30 s) was given along the direction perpendicular to the matrix plane. The penetration depth of DiD‐labeled nanomotors (blue) in the gel matrix was measured at 24 h after treatment.

### Cellular Uptake of Nanomotors

4T1 cells were seeded onto a 12‐well chamber slide at the density of 8 × 10^4^ cells per well. After culture overnight, cells were incubated with NP‐G/P for 2 h, 4 h, 8 h, and 12 h, respectively, and stained with DAPI followed by fixation with 4% parafomaldehyde. Then cells were observed under a confocal laser scanning microscope (CLSM, Zeiss, Germany). To quantify the cellular uptake of nanomotors, cells after nanomotor treatment were collected by trypsin digestion and subjected to flow cytometry (BD Biosciences, East Rutherford, USA).

### HIFU Enhanced Cytotoxicity of Nanomotors on Tumor Cells

4T1 cells were seeded onto a 96‐well plate at the density of 10^4^ cells per well and cultured overnight. To explore the stimulative effect of nanomotors on HIFU therapy, cells were treated with NP, NP‐PF or NP‐G/P (0.04 mg/mL of nanoparticles) for 12 h and then received 8.5 W HIFU treatment for different times. Afterward, cells were cultured for another 24 h and the cell viability was tested by the CCK‐8 assay. To explore the stimulative effect of HIFU irradiation on nanomotors, cells were treated with nanomotors at the GA concentration ranging from 0.1 to 10 µg mL^−1^ for 12 h and received 8.5 W HIFU treatment for 30 s. Afterward, after culture for another 24 h, the cell viability was tested by the CCK‐8 assay. Cells treated with NP‐G/P, or HIFU only were used as controls.

The apoptosis and necrosis of TNBC cells treated with HIFU‐driven nanomotors were measured by using Annexin V‐FITC/PI Cell Apoptosis Kit according to the manufacturer's protocol. Briefly, 4T1 cells treated with nanomotors (0.5 µg mL^−1^ of GA) for 12 h received 8.5 W HIFU treatment for 30 s and cultured for another 24 h. Afterward, cells were harvested by trypsin digestion and centrifugation, and stained with Annexin V‐FITC and propidium iodide (PI) for 15 min at room temperature in dark place. Then, cells were subjected a FACS Calibur flow cytometer (BD Biosciences, East Rutherford, USA) for flow cytometry analysis (FlowJo v.7.6.5, Tree Star, Inc., Ashland, USA). Cells treated with NP‐G/P or HIFU only were used as controls. To directly visualize the cell killing effect of HIFU‐driven nanomotors (NP‐G/P+H), 4T1 cells after treatment with HIFU‐driven nanomotors as described above were stained with Calcein‐AM and PI dispersed in PBS for 15 min. After washing with PBS for three times, cells were observed under a CLSM (Zeiss, Germany).

### Validation of Ferroptosis of TNBC Cells Trigged by HIFU‐Driven Nanomotors

To discover the specific cell‐killing mechanisms of HIFU‐driven nanomotors and validate the cell death pathway of TNBC cells after nanomotor treatment, the gene expression of tumor cells was determined through the RNA‐sequencing technique. In brief, 4T1 cells seeded onto 6‐well plates were cultured overnight, treated with NP‐G/P+H, and cultured for 12 h. Afterward, cells were collected for RNA extraction and RNA‐sequencing on a lllumina Novaseq 6000 (LC‐Bio Technology CO., Ltd., Hangzhou, China) as described above. Cells treated with NP‐G/P or HIFU only were used as controls. The differentially expressed genes that were significantly enriched in GO terms and metabolic pathway were performed by GO functional enrichment and KEGG pathway analysis.

Considering that thioredoxin (TRX) is the main target of GA, the TRX concentration in tumor cells after GA or NP‐G/P+H (0.5 µg/mL of GA) treatment was analyzed by ELISA assay. Briefly, 4T1 cells were seeded in 12‐well plates at a density of 1×10^5^ cells per well and cultured overnight. Afterward, cells were treated with GA or NP‐G/P for 8 h, irradiated with HIFU (8.5 W, 30 s) for the NP‐G/P+H group, and cultured for another 16 h. After that, cells were collected, lysed in an ice‐bath, and spin down, and the TRX or MCL1 concentration in the supernatant was analyzed by using the TRX or MCL1 ELISA assay kit according to the manufacturer's instructions. Cells treated with NP‐G/P or HIFU only were used as controls.

Then the cell ferroptosis after NP‐G/P+H treatment was validated by quantifying the concentration changes of ferroptosis‐related markers including intracellular ROS, GSH, LPO, and Fe^2+^. Briefly, 4T1 cells were planted onto 12‐well chamber slides and cultured overnight. Afterward, cells were treated with NP‐G/P (0.5 µg mL^−1^ of GA) for 8 h, irradiated with HIFU (8.5 W 30 s), and cultured for another 1 h. Cells were stained DCFH‐DA and DAPI, washed twice with PBS, and then subjected to a FACS Calibur flow cytometer (BD Biosciences, East Rutherford, USA) for ROS detection. Cells treated with NP‐G/P or HIFU only were used as controls. For the measurement of intracellular GSH, cells were treated with NP‐G/P (0.5 µg mL^−1^ of GA) for 8 h, irradiated with HIFU (8.5 W for 30 s), and cultured for another 16 h. Cells were then collected, lysed, and spin down by centrifugation. Afterward, the intracellular GSH content in the supernatant was examined by a GSH/GSSG assay kit. For the LPO measurement, cells were treated with NP‐G/P (0.5 µg mL^−1^ of GA) for 8 h, irradiated with HIFU (8.5 W for 30 s), and cultured for another 16 h. Cells were then incubated with C11‐BODIPY at 37 °C for 30 min and washed three times with PBS. Afterward, the fluorescence signal of LPO in cells was imaged under a CLSM (Zeiss, Germany) and analyzed through a FACS Calibur flow cytometer (BD Biosciences, East Rutherford, USA). For the detection of intracellular Fe^2+^, cells were treated with NP‐G/P (0.5 µg mL^−1^ of GA) for 8 h, irradiated with HIFU (8.5 W 30 s), and cultured for another 16 h. Cells were then stained with FerroOrange for 30 min, and analyzed with a FACS Calibur flow cytometer (BD Biosciences, East Rutherford, USA).

Tumor cell ferroptosis after NP‐G/P+H treatment was further validated by ferroptosis inhibitors. In brief, 4T1 cells were seeded in the 96‐well plate and cultured overnight. Afterward, cells were treated NP‐G/P+H (0.5 µg mL^−1^ of GA) for 24 h in the presence of different ferroptosis inhibitors including ferrostatin‐1 (100 nm), deferoxamine (100 µm), or vitamin E (20 µm). Then the cell viability after different treatments was assessed by the CCK‐8 method.

### Detection of DMPAs

4T1 cells were planted onto 12‐well chamber slides and cultured overnight. Afterward, cells were treated with NP‐G/P (0.5 µg mL^−1^ of GA) for 8 h, irradiated with HIFU (8.5 W 30 s), and cultured for another 12 h. The culture medium from each well was collected and used to detecting ATP by the ATP detection kit. Cells were then collected, lysed, and spin down by centrifugation. Afterward, the intracellular HMGB‐1 content in the supernatant was examined by the HMGB‐1 ELISA assay kit. To determine the calreticulin (CTR) level in 4T1 cells, cells were cultured and treated with NP‐G/P as described above. Afterward, cells were collected, incubated with anti‐calreticulin recombinant rabbit monoclonal antibody (diluted 1:2000) for 1 h and iFluor 488‐conjugated goat anti‐rabbit IgG antibody (diluted 1:1000) for 30 min successively, and subjected to flow cytometry (BD Biosciences, East Rutherford, USA).

### DC Maturation Induced by Nanomotor‐Treated Tumor Cells

The immune effect triggered by tumor cell ferroptosis was investigated in vitro. In brief, BMDCs were isolated from female BALB/C mice according to an established method,^[^ ^31^ ^]^ and then were cultured in RPMI‐1640 cell culture medium supplemented with GM‐CSF (20 ng mL^−1^) and IL‐4 (10 ng mL^−1^). After culture for 5 days, BMDCs were suspended in the fresh culture medium in the receptor well of 12‐well Transwell plates, were cocultured with NP‐G/P+H‐treated 4T1 cells which were seeded in the donor well. After coculture for 24 h, BMDCs were successively stained with anti‐CD11c‐FITC, anti‐CD80‐PE, anti‐CD86‐APC for DC maturation analysis by using flow cytometry (BD Biosciences, East Rutherford, USA). TNF‐α and IFN‐γ concentrations in the supernatant of receptor wells were measured by ELISA kits (Multisciences, China).

### Pharmacokinetics Study and Tissue Distribution

For building 4T1 solid tumor‐bearing mouse models, BALB/c mice received an orthotopic injection of 5 × 10^6^ 4T1 breast cancer cells on the right mammary gland. When the tumor volume reached ≈100 mm^3^, tumor‐bearing mice were intravenously administered with 200 µL of DiD‐NP or DiD‐NP‐G/P at the DiD dose of 2 µg. To assess the pharmacokinetics of nanomotors, 20 µL of blood was collected from the eye socket of mice at different time points after injection and mixed with 20 µL of EDTA‐Na_2_ solution (100 U mL^−1^). Afterward, the fluorescence intensity of blood samples was quantified with a Tecan microplate reader (Ex/Em = 640/670 nm, Switzerland). The concentration of nanomotors in the blood was expressed as ID%/mL (Percentage of injected dose per mL of blood). To directly visualize the tumor accumulation of HIFU‐driven nanomotors, mice received HIFU irradiation (8.5 W for 30 s) at the tumor site at 3 h after DiD‐NP‐G/P injection and imaged with the IVIS Spectrum live‐imaging system (PerkinElmer, USA) at different time points after injection. At 24 h after intravenous administration, mice were sacrificed to collect major organs and tumors for *ex vivo* imaging with the IVIS Spectrum live‐imaging system. After imaging, they were suspended in PBS, homogenized, and subjected to a fluorescence spectrophotometer (Biotek Synergy H1, USA) for measuring the fluorescence intensity (Ex/Em = 640/670 nm). The concentration of nanomotors in the organs was expressed as ID%/g (Percentage of injected dose per gram of tissue). To visualize the penetration pattern of nanomotors in tumors, mice were sacrificed to collect tumors at 12 h after injection for preparing frozen slices. After staining with DAPI, tumor slices were scanned with a Pannoramic P‐MIDI (3DHISTECH Ltd, Hungary).

### Improved Antitumor Efficacy of NP‐G/P Driven by HIFU on TNBC Models

TNBC model mice were randomly divided into 4 groups 7 days post tumor inoculation, and received an intravenous injection of NP‐G/P at the GA dose of 12 µg or an equal volume of PBS every other day for two times. Three hours after each injection, mice were anesthetized by isoflurane and received 8.5 W HIFU irradiation for 30 s at the tumor site. At 24 h after the second injection, three mouse models in each group were sacrificed and tumors were collected for tissue section followed by H&E and TUNEL staining according to routine protocols. Tumor volumes and mouse weight in different treatment groups were recorded for 30 days after tumor inoculation. Mouse survival within 45 days was also monitored after tumor inoculation. Specifically, a tumor volume of more than 1500 mm^3^ was considered as the threshold for identifying death. Mouse models treated with NP‐G/P or PBS only were used as controls. To confirm the tumor ferroptosis induced by HIFU‐driven nanomotors, 4T1‐bearing tumor mice were randomly divided into five groups including the PBS, NP‐G/P, HIFU, NP‐G/P+H, NP‐G/P+H+Fer‐1 groups 7 days post tumor inoculation and received an intravenous injection of NP‐G/P (12 µg of GA), NP‐G/P+ Fer‐1 (12 µg of GA and 16 µg of Fer‐1), or an equal volume of PBS every other day for two times. Three hours after each injection, mice were anesthetized and received HIFU irradiation as described above. At 3 days after the last injection, 3 mouse models in each group were sacrificed, and tumors were collected and sectioned for immunofluorescence staining of LPO with C11‐BODIPY following the manufacturer's procedure. Tumor slices were then scanned with a Pannoramic P‐MIDI (3DHISTECH Ltd, Hungary)

### Anti‐Tumor Immunity Induced by HIFU‐Driven Motors on TNBC Models

To investigate the antitumor immune response, the tumor‐draining lymph nodes (TDLNs) and tumors were collected at day 7 after different treatments. The TDLNs were processed into single‐cell suspension. The suspended cells from TDLNs were successively stained with anti‐CD11c‐FITC, anti‐CD80‐PE, and anti‐CD86‐APC, and analyzed by flow cytometry (BD Biosciences, East Rutherford, USA) for the assessment of DC maturation. Tumors were dissociated with 10× Enzyme stock solution to produce a single‐cell suspension. Infiltrated T lymphocytes in tumors were stained with anti‐CD3‐PerCP, anti‐CD4‐FITC, and anti‐CD8‐AF647, and analyzed by flow cytometry (BD Biosciences, East Rutherford, USA). Tumors were also sectioned for staining with anti‐CD4‐FITC, anti‐CD8‐AF647, anti‐IL‐1 beta antibody, and anti‐thioredoxin antibody following the manufacturer's procedure and observed under a Pannoramic P‐MIDI (3DHISTECH Ltd, Hungary). In addition, the levels of TNF‐α and IFN‐γ in the tumor were measured by an ELISA kit according to the standard protocols.

### Anti‐Metastasis Efficacy Induced by HIFU‐Driven Nanomotors on TNBC Models

TNBC model mice were constructed and received different treatments as described above. At 3 days after the last nanomotor treatment, 4T1‐Luc cancer cells were intravenously injected into tumor‐bearing mice of different groups to create tumor metastases in the lung. The progress of lung metastases was monitored by bioluminescence imaging with the IVIS Spectrum live‐imaging system (PerkinElmer, USA) at different time points. Mice were intraperitoneally injected with 150 mg kg^−1^ of D‐luciferin potassium salt and imaged at 15 min after D‐luciferin injection. At the end of the experiment, mice were sacrificed and lungs were collected, fixed with Bouin's fixative solution. The metastatic lesions appearing as lung nodes were photographed and counted. Afterward, lungs were embedded in paraffin, sectioned, stained with H&E, and scanned with SLIDEVIEW VS200 (Olympus, Japan).

### Statistical Analysis

All the data were expressed as mean±standard deviation (SD). The difference between two groups was analyzed using the Student's *t*‐test. One‐way analysis of variance (ANOVA) followed by Tukey's *post hoc* test was used to determine differences between multiple groups. The statistical analyses of all data were conducted using GraphPad Prism 8.0. Bioinformatics analysis, data interpretation, and graph visualization were performed using R v4.1.2 (https://cran.r‐project.org/, R development core team).

## Conflict of Interest

The authors declare no conflict of interest.

## Supporting information

Supporting Information

## Data Availability

The data that support the findings of this study are available in the supplementary material of this article.
