# Supplementary material for: High Intensity Focused Ultrasound‐Driven Nanomotor for Effective Ferroptosis‐Immunotherapy of TNBC
Source: Adv Sci (Weinh). 2024 Feb 11;11(15):2305546. doi: 10.1002/advs.202305546 (PMC11022700; doi:10.1002/advs.202305546)
Supplement: Supplementary file 1 — Supporting Information [file ADVS-11-2305546-s001.pdf]

ADVANCED SCIENCE
Open Access

Supporting Information

for *Adv. Sci.*, DOI 10.1002/advs.202305546

High Intensity Focused Ultrasound-Driven Nanomotor for Effective Ferroptosis-Immunotherapy of TNBC

Xiangrong Yu, Xuejing Li, Qingwang Chen, Siyu Wang, Ruizhe Xu, Ying He, Xifeng Qin, Jun Zhang, Wuli Yang, Leming Shi, Ligong Lu, Yuanting Zheng*, Zhiqing Pang* and Shaojun Peng**

Supporting Information

High Intensity Focused Ultrasound-driven Nanomotor for Efective Ferroptosis-immunotherapy of TNBC

*Xiangrong Yu, Xuejing Li, Qingwang Chen, Siyu Wang, Ruizhe Xu, Ying He, Xifeng Qin, Jun Zhang, Wuli Yang, Leming Shi, Ligong Lu, *Yuanting Zheng,* Zhiqing Pang,* Shaojun Peng**

(Xiangrong Yu, Xuejing Li and Qingwang Chen contributed equally to this work)

Xiangrong Yu, Ligong Lu, Shaojun Peng

Guangdong Provincial Key Laboratory of Tumor Interventional Diagnosis and Treatment Zhuhai People's Hospital (Zhuhai Hospital Affiliated with Jinan University)

Zhuhai, Guangdong 519000, P. R. China

E-mail: luligong1969@jnu.edu.cn; shaojunpeng@ext.jnu.edu.cn

Xuejing Li, Siyu Wang, Ruizhe Xu, Ying He, Xifeng Qin, Zhiqing Pang

Key Laboratory of Smart Drug Delivery, School of Pharmacy,

Fudan University,

Shanghai, 201203, P.R. China

E-mail: zqpang@fudan.edu.cn

Qingwang Chen, Leming Shi, Yuanting Zheng

State Key Laboratory of Genetic Engineering, School of Life Sciences and Human Phenome Institute

Fudan University, 2005 Songhu Road,

Shanghai 200438, P.R. China

E-mail: zhengyuanting@fudan.edu.cn

Jun Zhang

Department of Radiology, Huashan Hospital, State Key Laboratory of Medical Neurobiology

Fudan University, 12 Wulumuqi Middle Road

Shanghai 200040, China

Wuli Yang

State Key Laboratory of Molecular Engineering of Polymers & Department of Macromolecular Science

Fudan University

Shanghai 200433, China

Methods

Cytotoxicity of GA on 4T1 cells

4T1 cells were seeded onto a 96-well plate at the density of 104 cells per well and cultured overnight. Afterward, cells were incubated with various concentrations of GA for 24 h and the cell viability was tested by the CCK-8 assay.

Considering that thioredoxin (TRX) is the main target of GA, the TRX concentration in tumor cells after GA treatment was analyzed by ELISA assay. Briefly, 4T1 cells were seeded in 12-well plates at a density of 1×10^5 cells per well and cultured overnight. Afterward, cells were treated with GA (1.0 μg/mL) for 24 h. After that, cells were collected, lysed in an ice-bath, and spin down, and the TRX concentration in the supernatant was analyzed by using the TRX ELISA assay kit according to the manufacturer's instructions. Cells treated with PBS was used as control.

Ex vivo cavitation ability of nanomotors under HIFU irradiation

The pork liver models were prepared in degassed water and received an injection of 100 μL of NP-G/P (5 mg/mL).[32] Pork livers injected with an equal volume of PBS or NP (5 mg/mL) served as controls. Then, the injection site was exposed to HIFU irradiation at 8.5W for 30 s. The cross-sectional digital images of the treated pork livers cut along the direction of HIFU irradiation were recorded and the radiation volume of HIFU was measured by the following formula: $Volume=\pi/4\times W^2\times D$ (W: width, D: depth).

Primary assessment of the biosafety

Healthy BABL/c mice and TNBC-bearing mice were sacrificed at the end of the experiment, and the major organs including the heart, liver, spleen, lung, and kidneys were excised and embedded in paraffin. Then these tissues were sectioned for hematoxylin and eosin (H&E) staining to observe the histological changes. The blood was also collected to perform the routine blood test, including the ratio of lymph, the concentrations of RBCs, white blood cells (WBCs), hemoglobin (HGB), platelets (PLTs), mean corpuscular hemoglobin (MCH), mean corpuscular volume (MCV), and red cell volume distribution width (RDW). Blood samples were collected from healthy mice for blood biochemical analysis, including measurements of aspartate aminotransferase (AST), alkaline phosphatase (ALP), alanine transaminase (ALT), creatinine (CRE), and blood urea nitrogen (BUN).

Statistical analysis

All the data were expressed as mean ± standard deviation (SD). The difference between two groups was analyzed using the Student's *t*-test. One-way analysis of variance (ANOVA) followed by Tukey's *post hoc* test was used to determine differences between multiple groups.

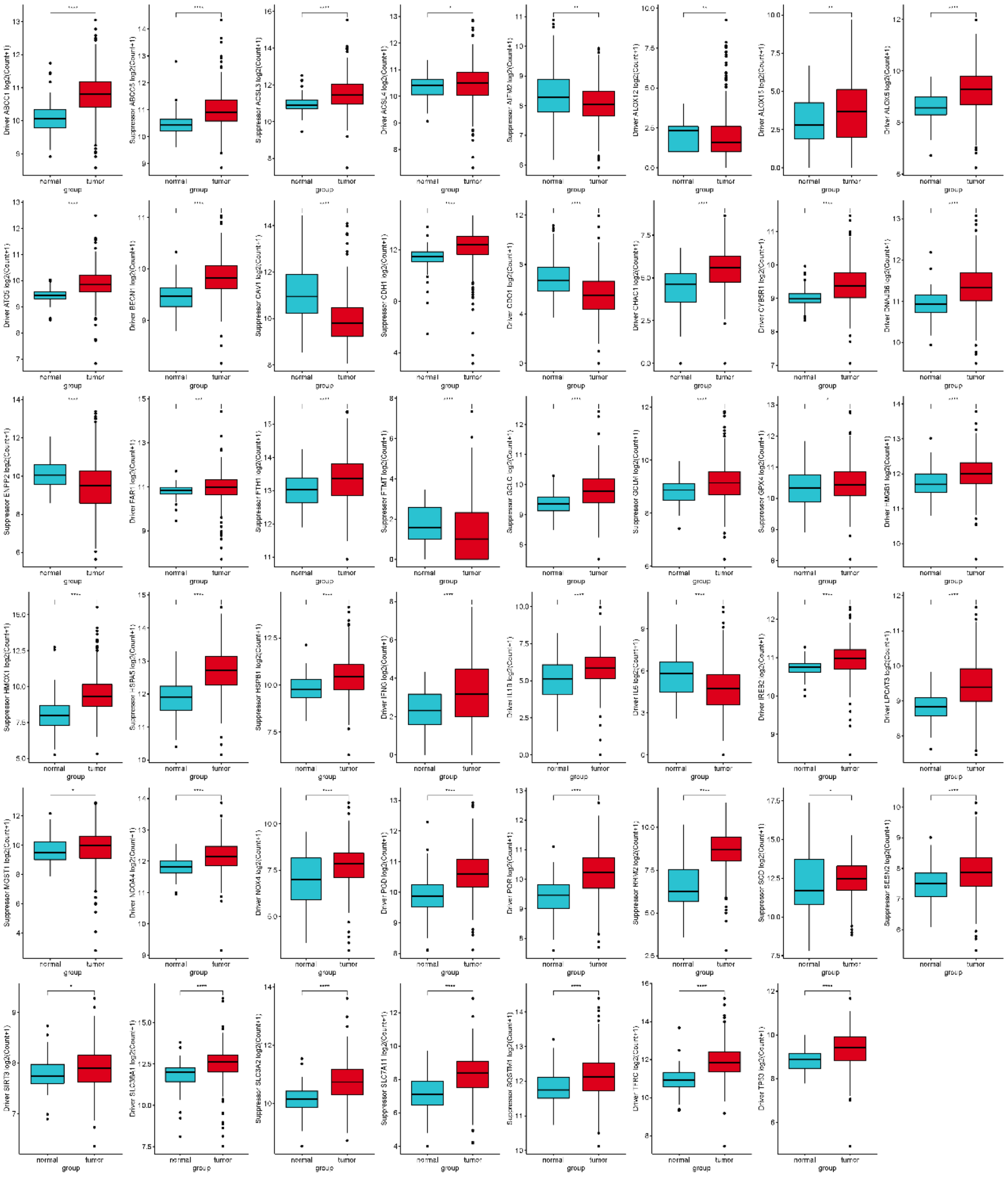

Figure S1. Box plots of the expression levels of 47 typical ferroptosis-associated genes in TNBC and normal breast tissues from FUSCCTNBC cohort. **P*< 0.05, ***P*< 0.01, ****P*< 0.001, and *****P*< 0.0001.

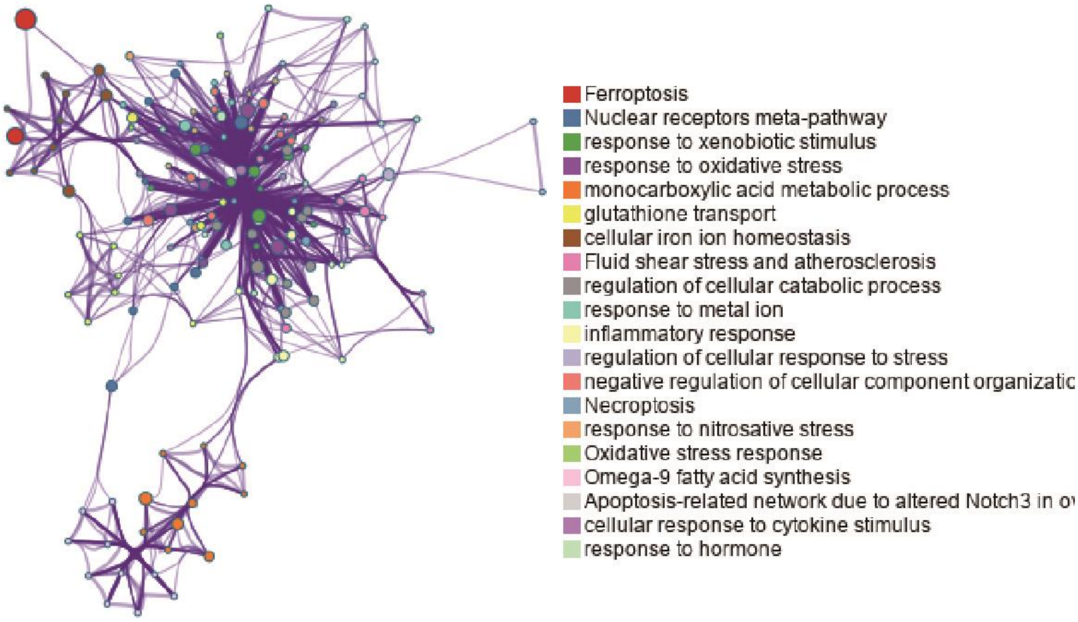

Figure S2. Gene Ontology (GO) enrichment analysis of 47 typical ferroptosis-associated genes.

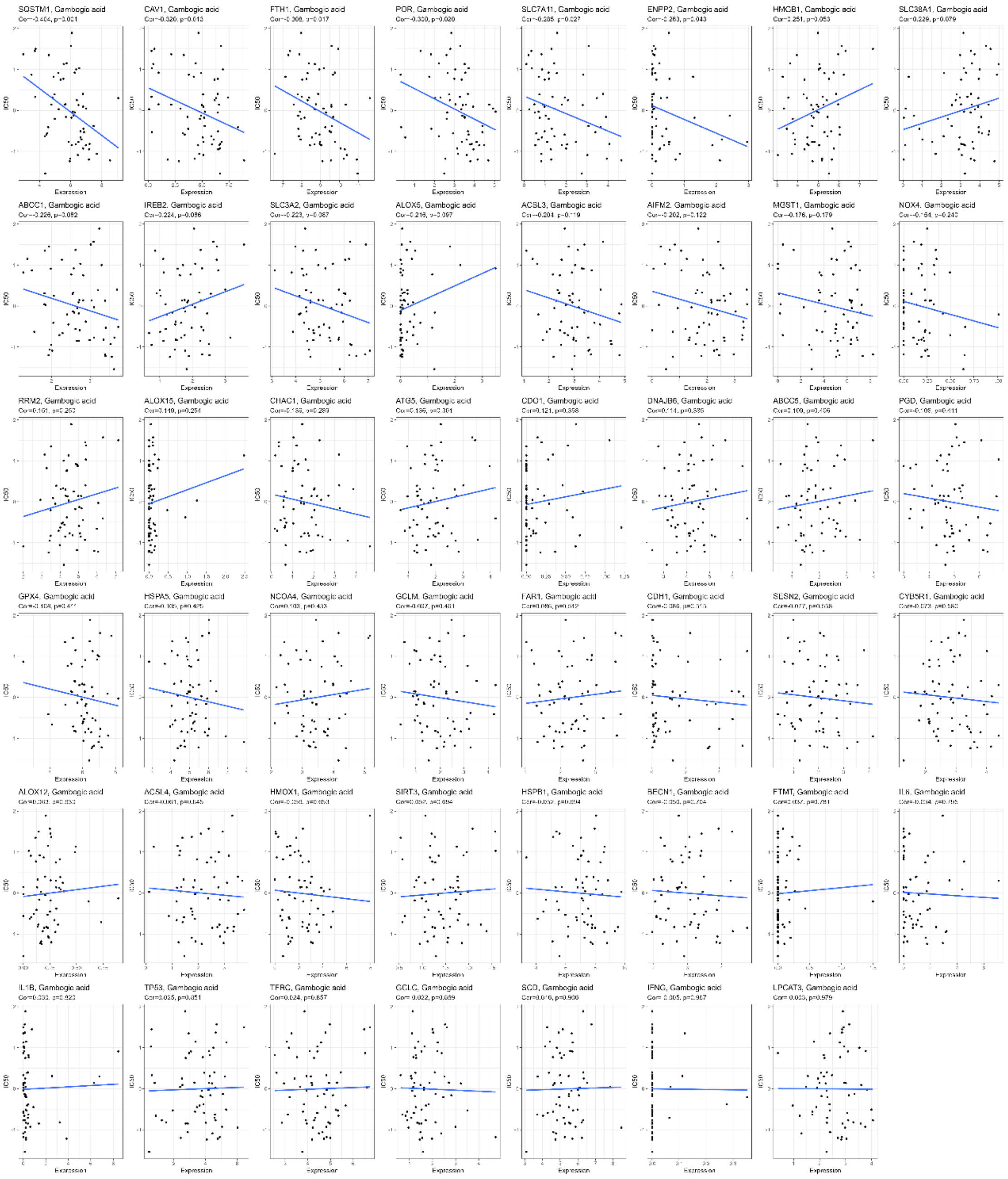

Figure S3. The scatter plots showed the correlation between the expression levels of ferroptosis-related genes and the IC50 value of GA.

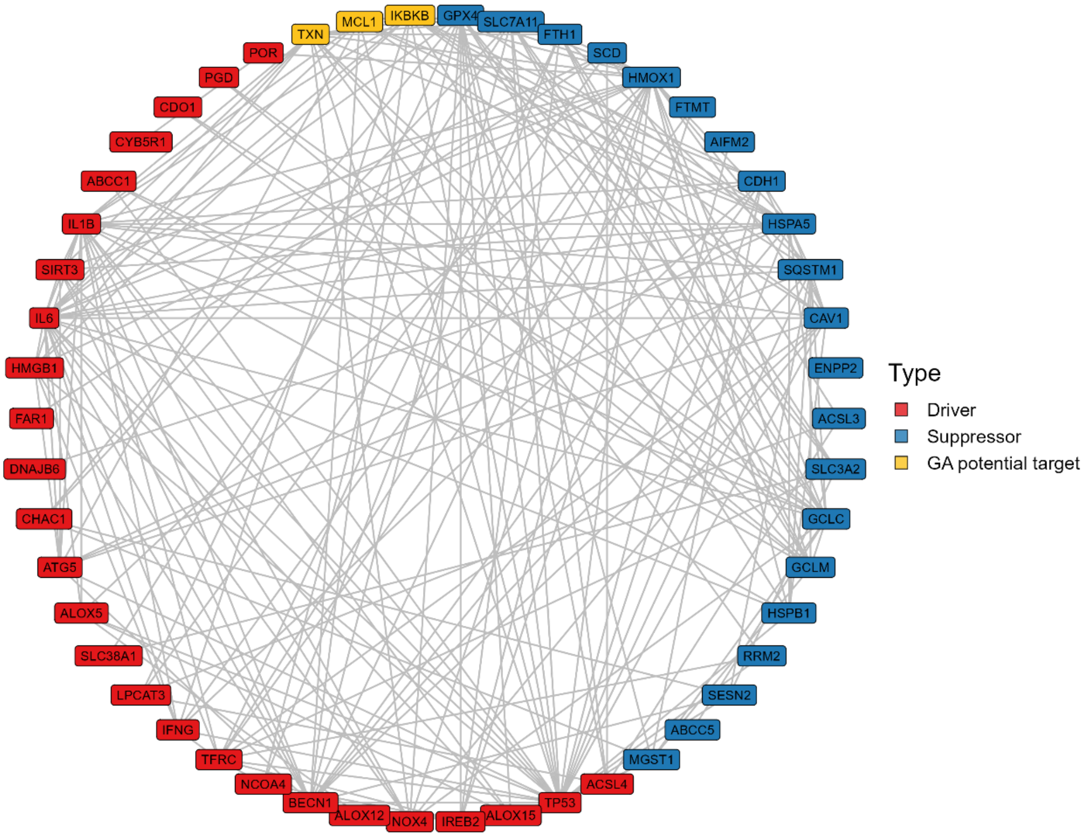

Figure S4. Protein-protein interaction (PPI) network of potential target proteins and ferroptosis regulators.

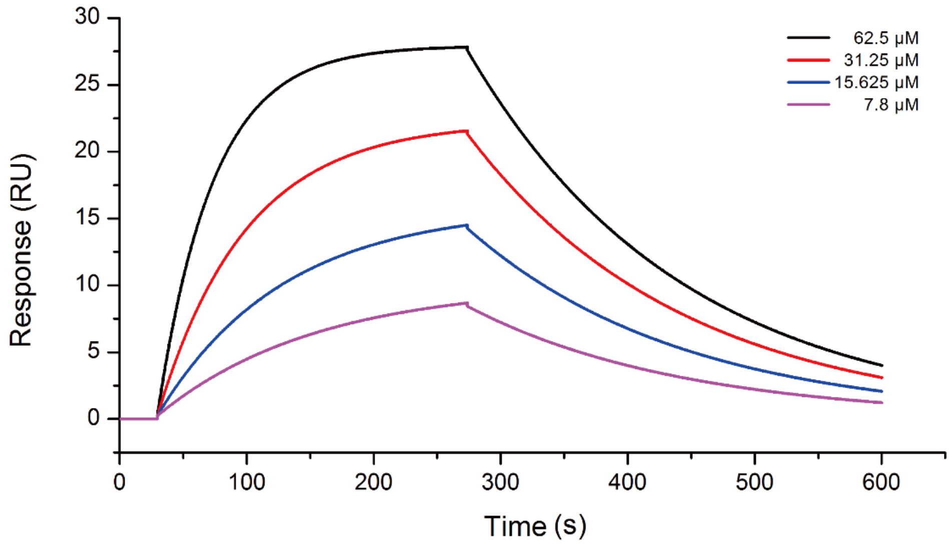

Figure S5. Surface plasmon resonance analysis of the affinity between GA and thioredoxin (TRX).

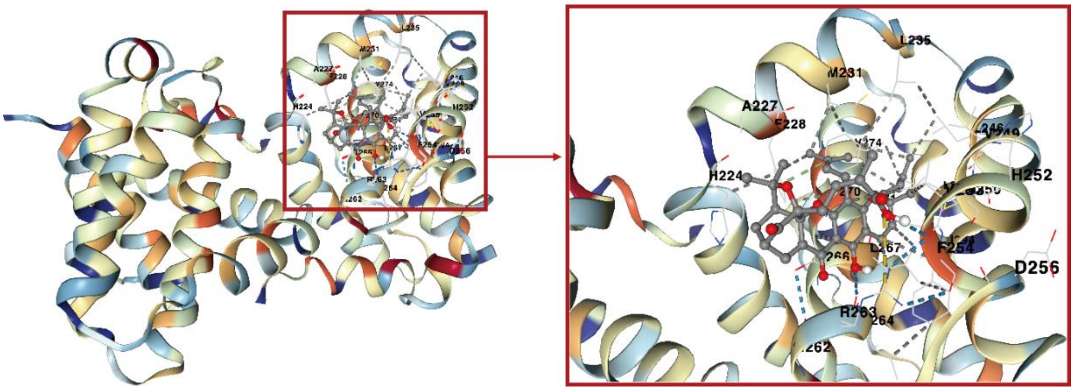

Figure S6. Structures of the MCL1 protein and GA combination predicted with crystal molecular docking.

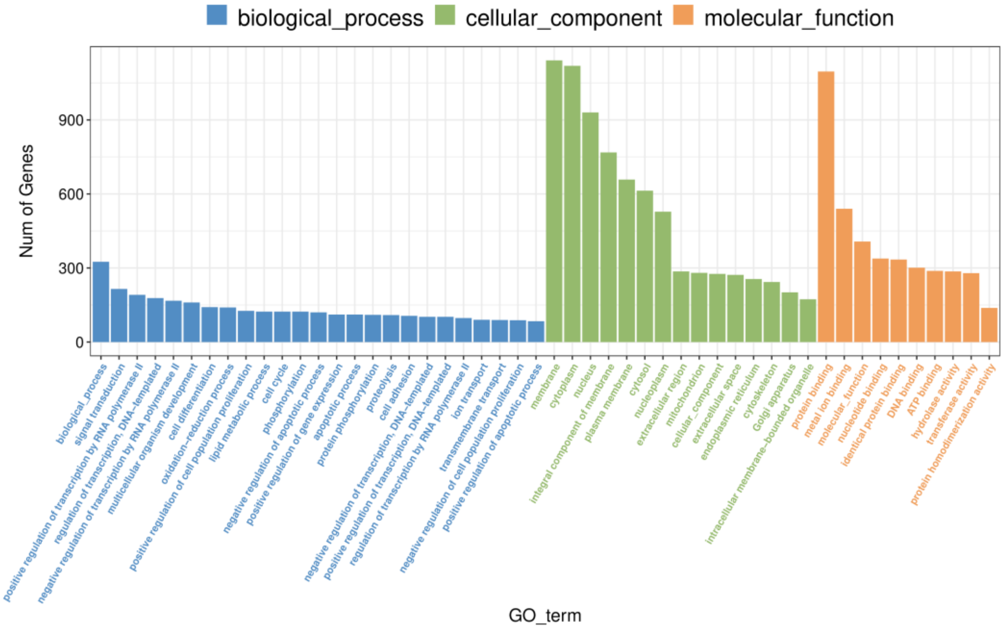

Figure S7. GO enrichment analysis of gene expression in GA-treated 4T1 cells.

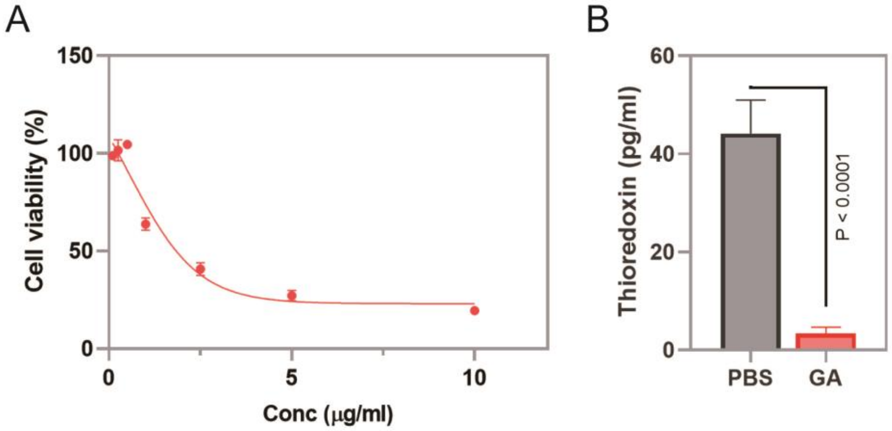

Figure S8. (A) The cell viability of 4T1 cells after GA treatment determined by the Cell-Counting Kit 8 (CCK-8) (n=3). **(B)** The intracellular concentration of thioredoxin (TRX) in GA-treated 4T1 cells (n=3).

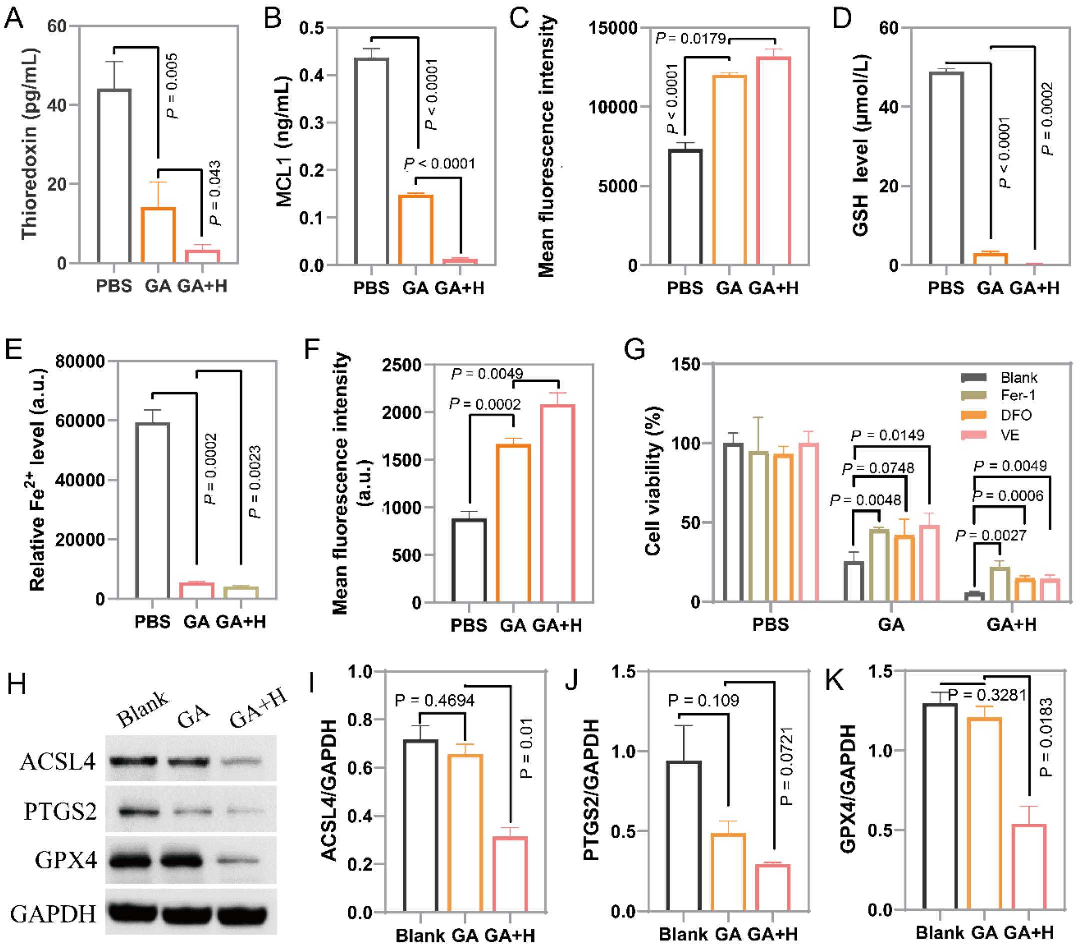

Figure S9. (A, B) The intracellular thioredoxin **(A)** and MCL1 **(B)** concentrations in 4T1 cells after different treatments (n=3). **(C)** Relative ROS levels in 4T1 cells after various treatments. **(D)** Intracellular GSH concentration in 4T1 cells after various treatments (n=3). **(E)** The relative expression level of Fe (II) in 4T1 cells after various treatments (n=3). **(F)** The intracellular LPO level in 4T1 cells after various treatments (n=3). **(G)** The cell viability of 4T1 cells that received different treatments in the presence of different ferroptosis inhibitors including Fer-1, DFO, and vitamin E (n=3). Western blot analyses of ACSL4, PTGS2, and GPX4 expressions (**H)** in 4T1 cells after GA and GA+H treatments and the corresponding semi-quantitation results **(I-K).**

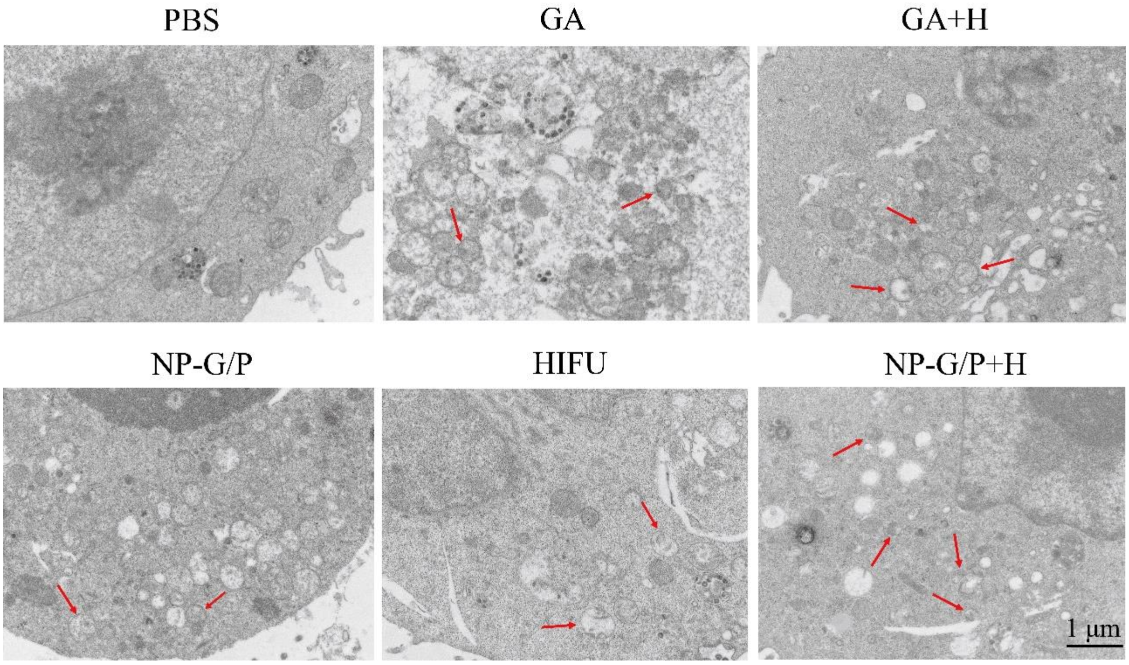

Figure S10. Identification of changes in mitochondrial features in 4T1 cells after different treatments through TEM imaging. Red arrowheads, ferroptotic mitochondria.

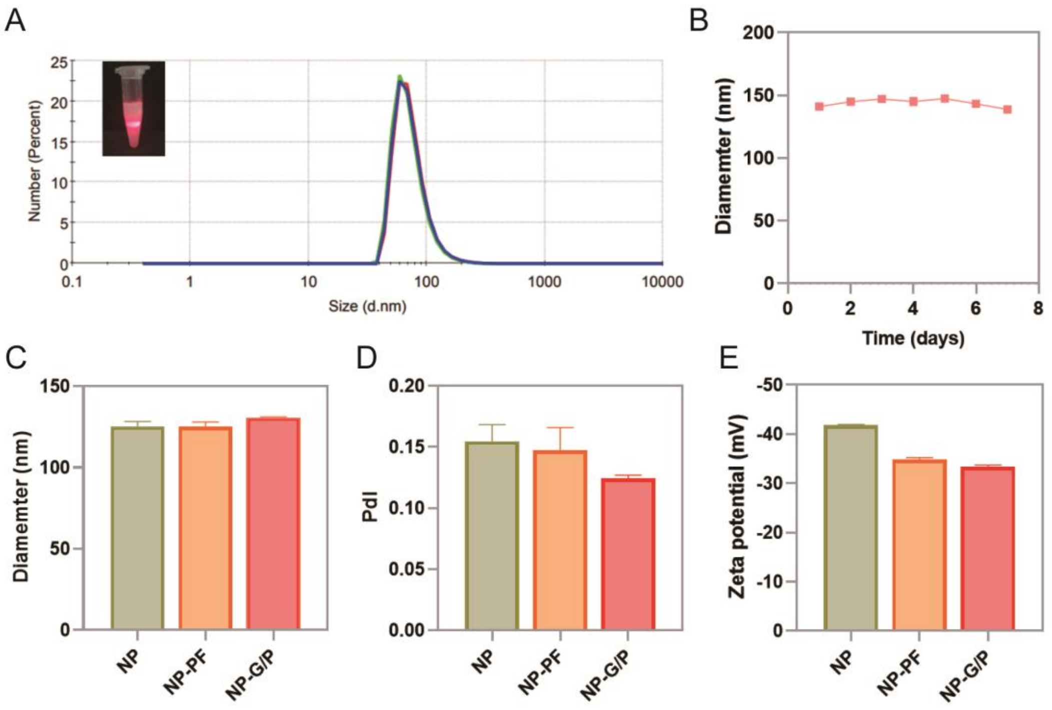

Figure S11. Characterization of nanomotors. (**A and B**) The number-based size distribution (**A**) and the mean size (**B**) of NP-G/P stored in PBS at 4 °C for 7 days (n=3). (**C to E**) The mean size (**C**), polydisperse index (PdI) (**D**), and zeta potential (**E**) of different nanoparticles (n=3).

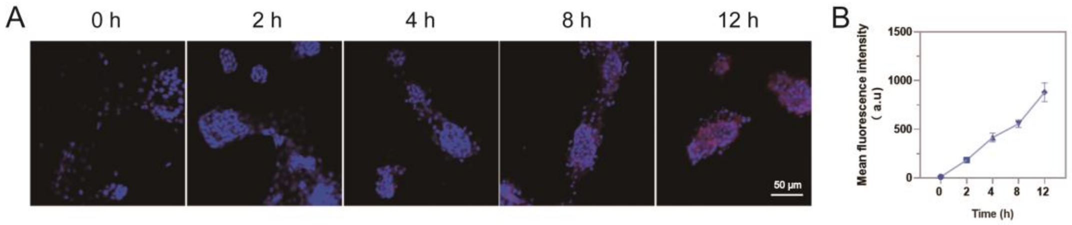

Figure S12. Cellular uptake of NP-G/P by 4T1 cells. (**A**) Representative fluorescence images of 4T1 cells treated with DiD-labeled NP-G/P for different times (n=3). (**B**) the fluorescence intensity of 4T1 cells treated with DiD-labeled NP-G/P for different times was determined by flow cytometry (n=3).

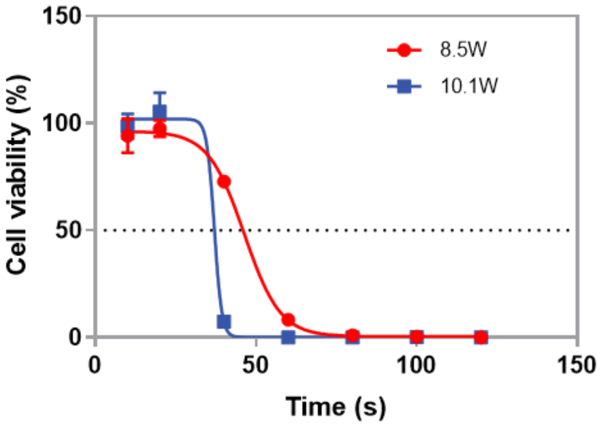

Figure S13. The cell viability of 4T1 cells after HIFU treatment with different powers and irradiation times determined by the CCK-8 method (n=3).

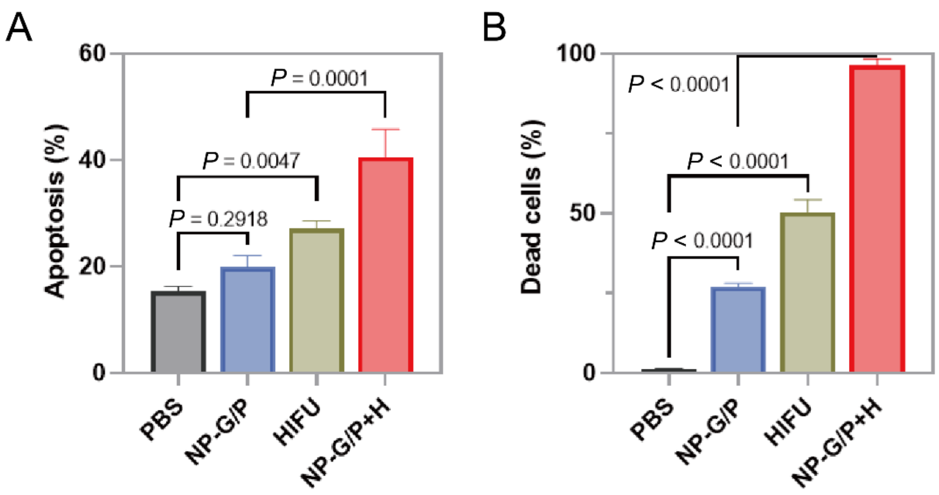

Figure S14. Cytotoxicity of HIFU-driven nanomotors *in vitro*. (A) Apoptosis analysis of 4T1 cells after different treatments determined by flow cytometry (n=3). (B) Live/dead cell assay of 4T1 cells after different treatments (n=3).

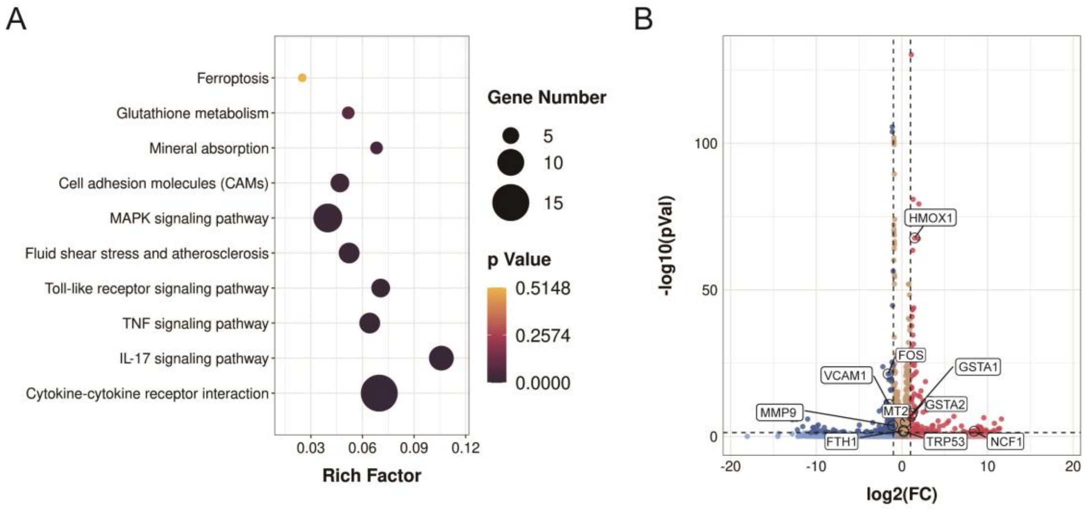

Figure S15. (**A**) The top 10 pathways in which differentially expressed genes of the NP-G/P+H group versus the NP-G/P group in 4T1 cells were enriched by the KEGG enrichment analysis. (**B**) The volcano map showed the upregulated and downregulated genes in 4T1 cells after NP-G/P+H treatment compared with PBS treatment. The screening criteria were FDR<0.05 and $|\log_2$fold change$|>=1$.

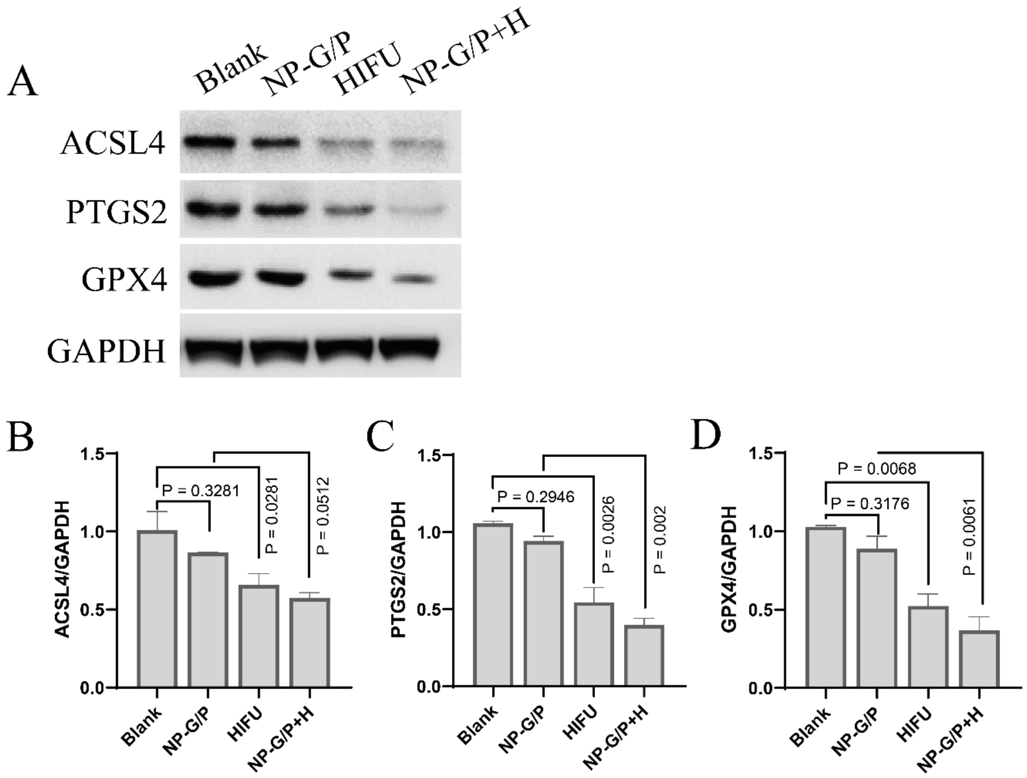

Figure S16. Western blot analyses of ACSL4, PTGS2, and GPX4 expression (**A**) in 4T1 cells after different treatments and the corresponding semi-quantitation results (**B-D**).

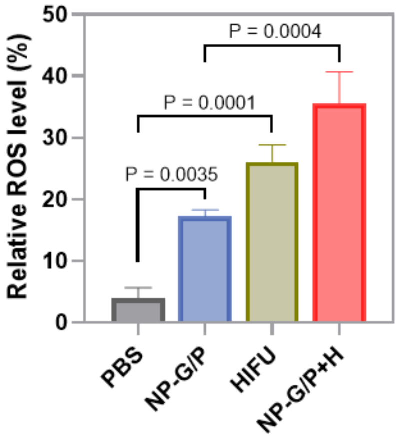

Figure S17. Relative intracellular ROS level in 4T1 cells after different treatments determined by flow cytometry (n=3).

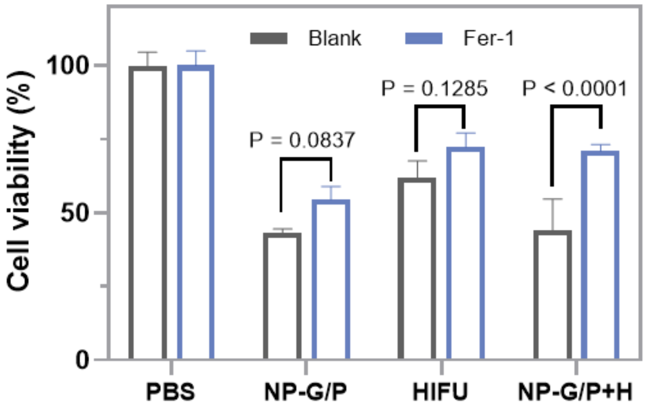

Figure S18. The viability of 4T1 cells after different treatments in the presence of Fer-1 determined by the CCK-8 method (n=3).

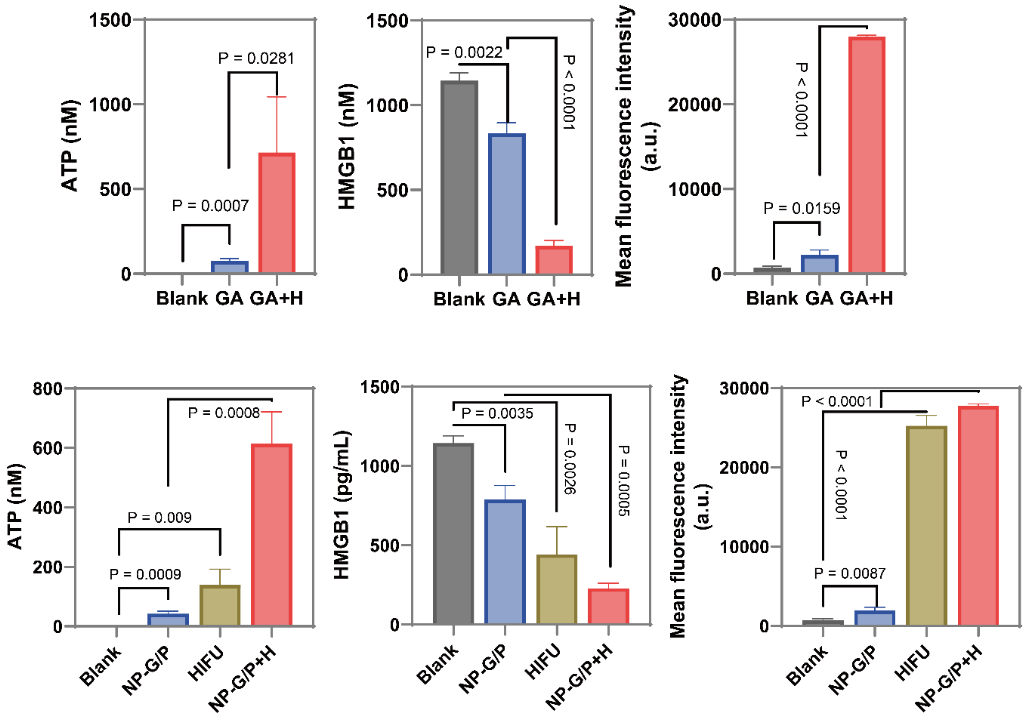

Figure S19. Extracellular ATP level, intracellular HMGB-1 level, and average fluorescence intensity of CRT from 4T1 cells after different treatments (n=3).

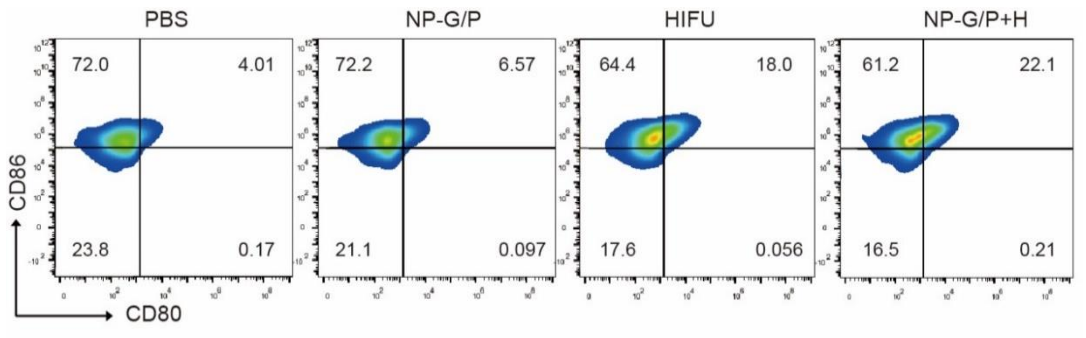

Figure S20. Representative flow cytometry charts of CD80$^+$CD86$^+$ BMDC cells in different groups.

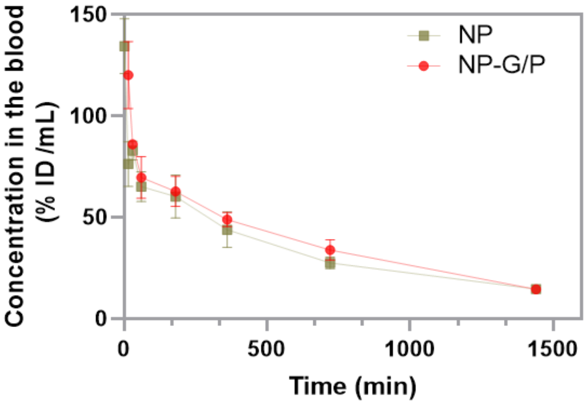

Figure S21. Concentration-time profiles of different formulations (NP and NP-G/P) in the mouse plasma after injection (n=3).

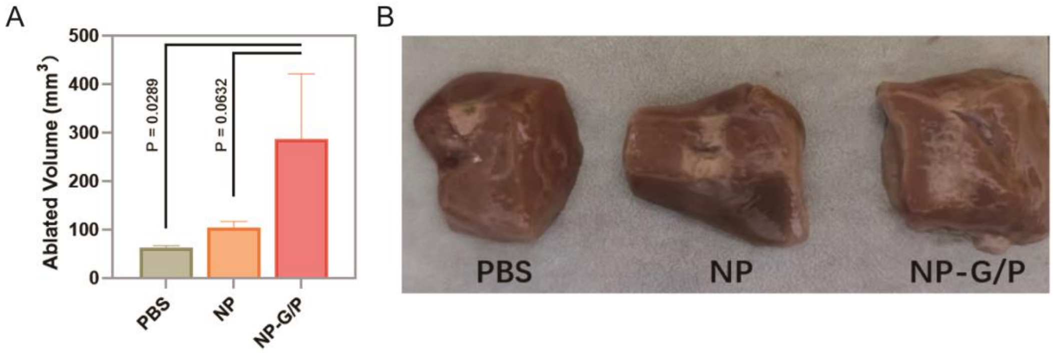

Figure S22. *Ex vivo* evaluation of the cavitation effect of nanomotors. (**A**) The radiation volume of pork livers injected with PBS, NP, or NP-G/P after HIFU irradiation at 8.5 W for 30 s (n=3). (**B**) Representative photographs of pork livers cut along the direction of HIFU irradiation.

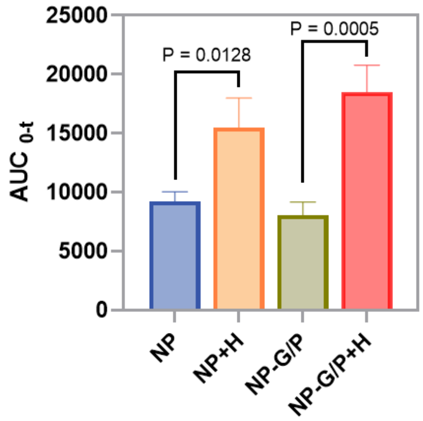

Figure S23. The estimated AUC of nanomotors at the tumor site based on *in vivo* fluorescence imaging (n=3).

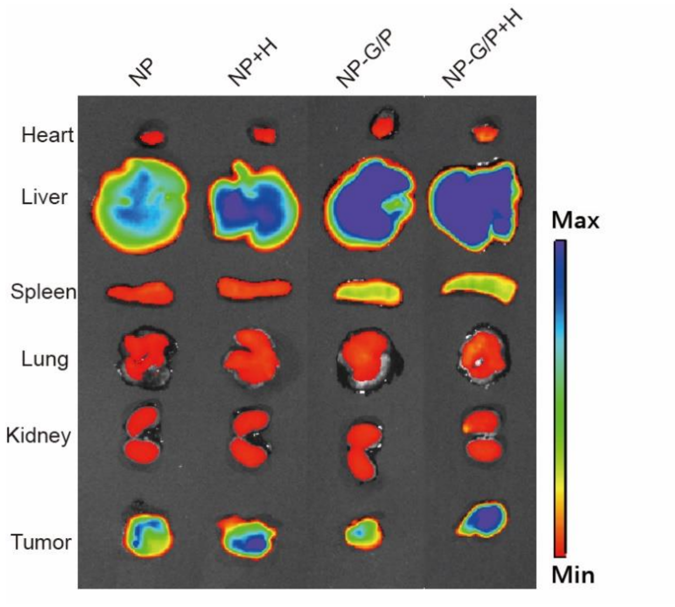

Figure S24. Representative *ex vivo* fluorescence images of major organs and tumors collected at 24 h after injection of nanomotors.

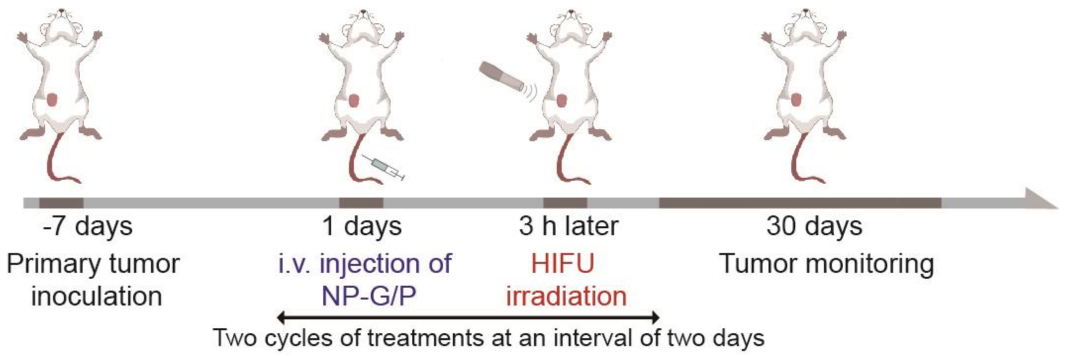

Figure S25. Schematic diagram of the experiment design using NP-G/P+H to treat mice bearing 4T1 tumors.

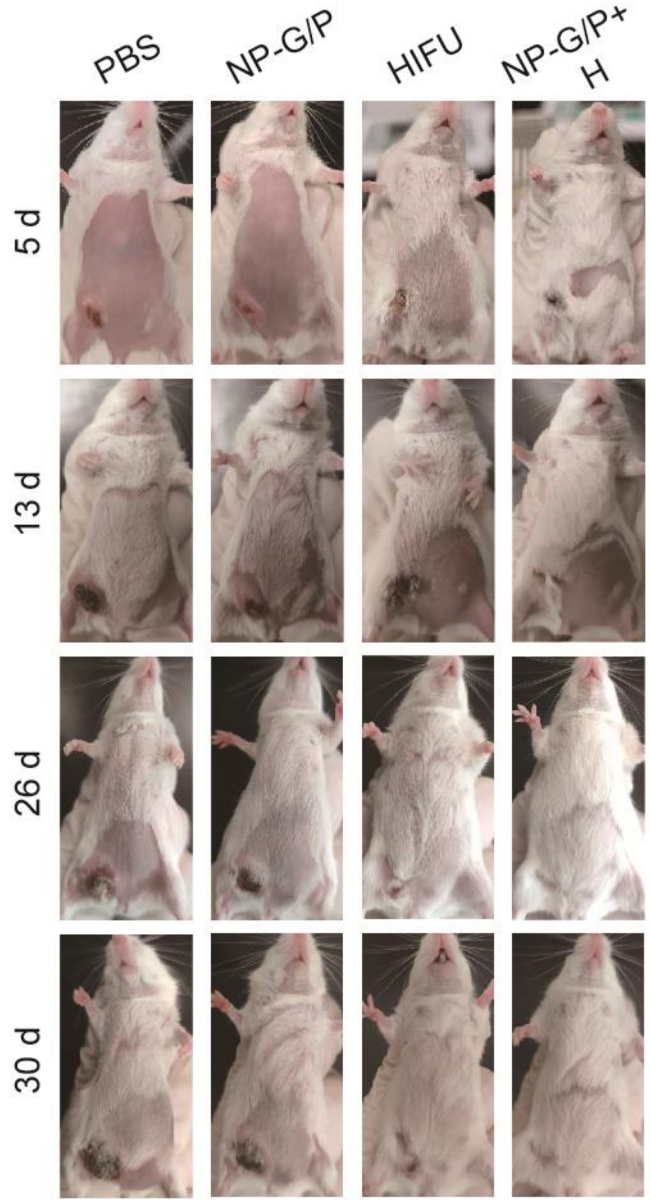

Figure S26. Representative photographs of primary tumors in the different treatment groups on Day 5, 13, 26, and 30.

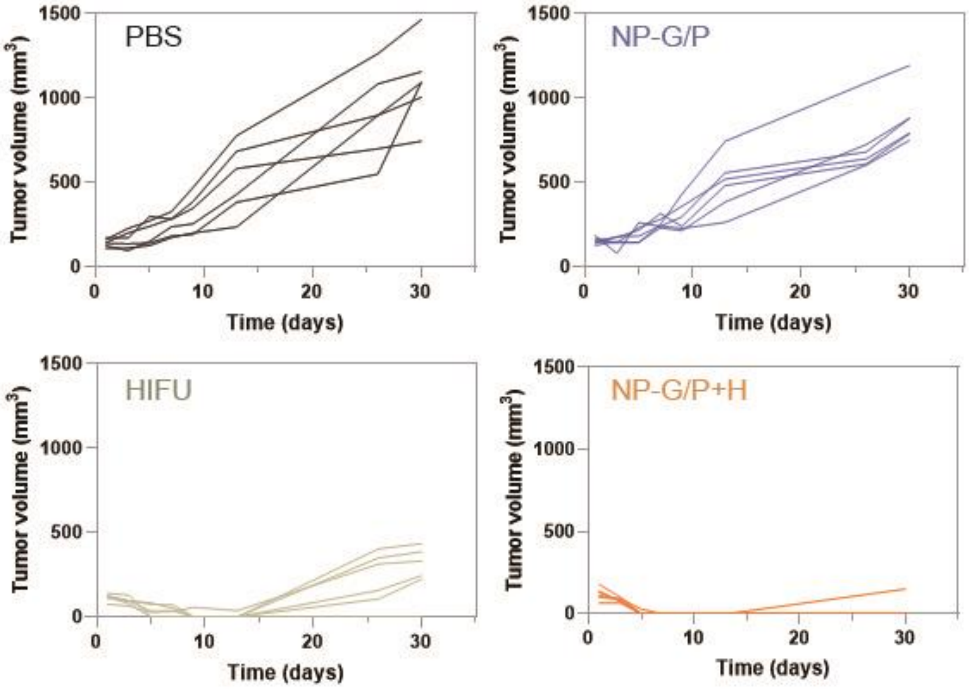

Figure S27. Tumor growth curves of primary tumors from individual mouse in different treatment groups (n=6).

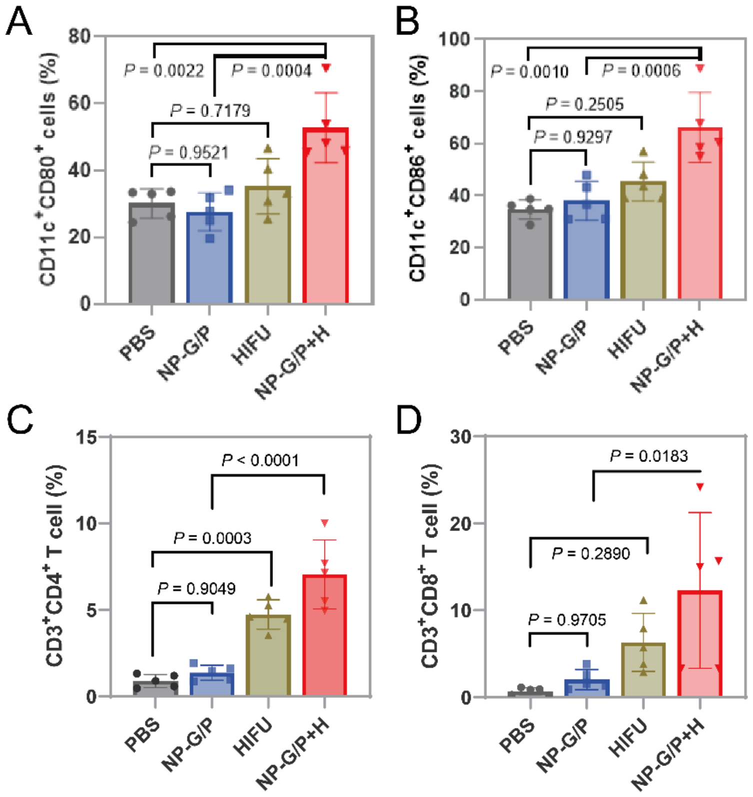

Figure S28. The antitumor immunity induced by HIFU-driven nanomotors on TNBC models. (**A,B**) Percentages of $CD80^+$ (**A**) and $CD86^+$ (**B**) in $CD11c^+$ DCs from the tumor-draining lymph nodes after different treatments detected by flow cytometry (n=5). (**C,D**) Percentages of $CD4^+$ (**C**) and $CD8^+$ (**D**) T cells in primary tumors after different treatments detected by flow cytometry (n=5).

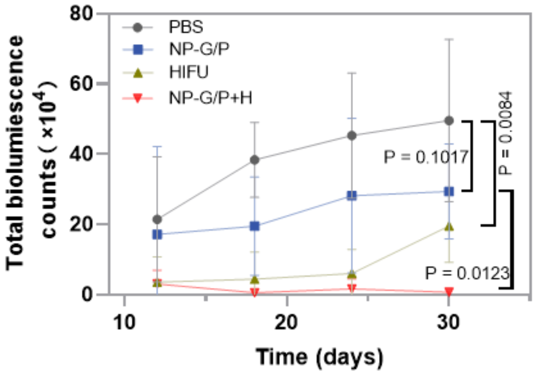

Figure S29. Bioluminescence intensity of mice at different time points after various treatments (n=5).

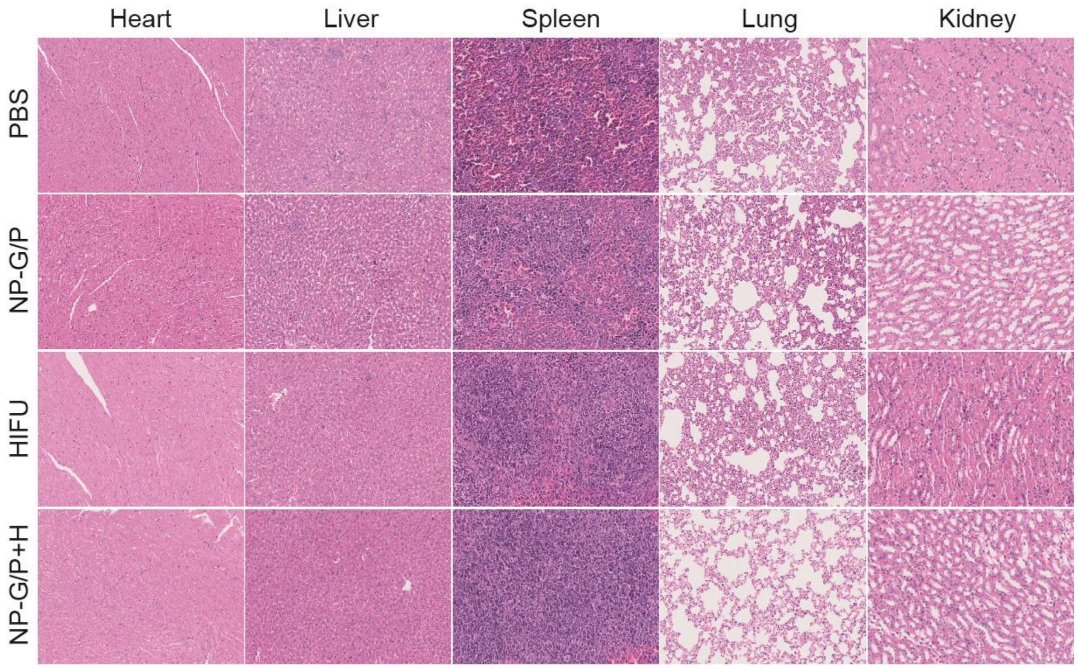

Figure S30. Representative images of H&E-stained slices from major organs after different treatments.

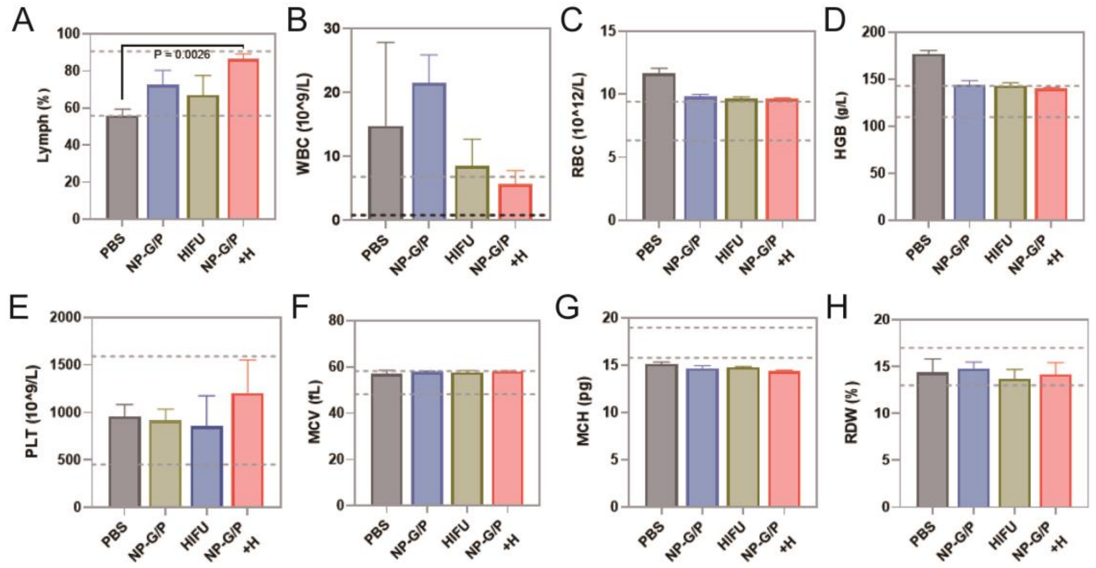

Figure S31. Hematology data in 4T1 tumor-bearing BALB/c mice after various treatments (n=3).

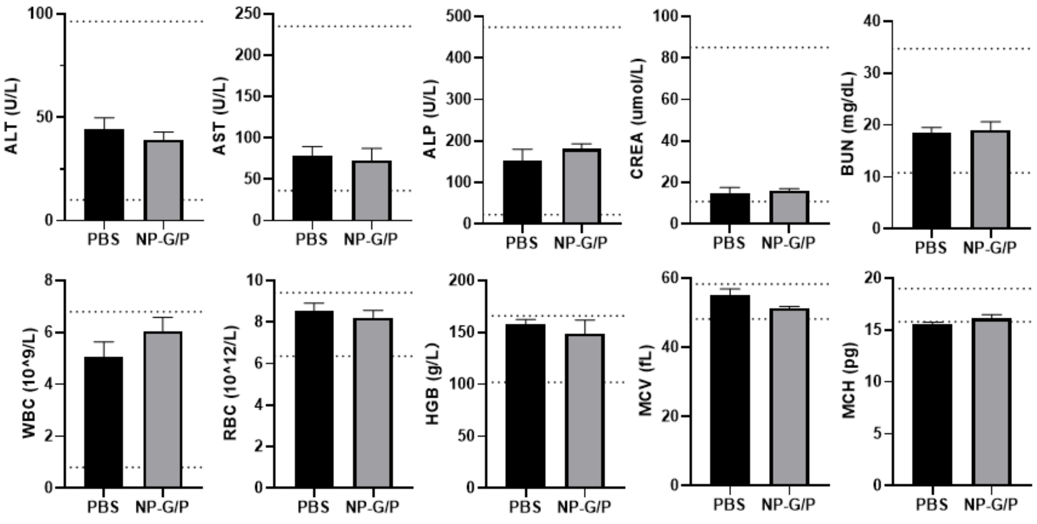

Figure S32. Hematology data in healthy BALB/c mice after PBS and NP-G/P treatments (n=3).

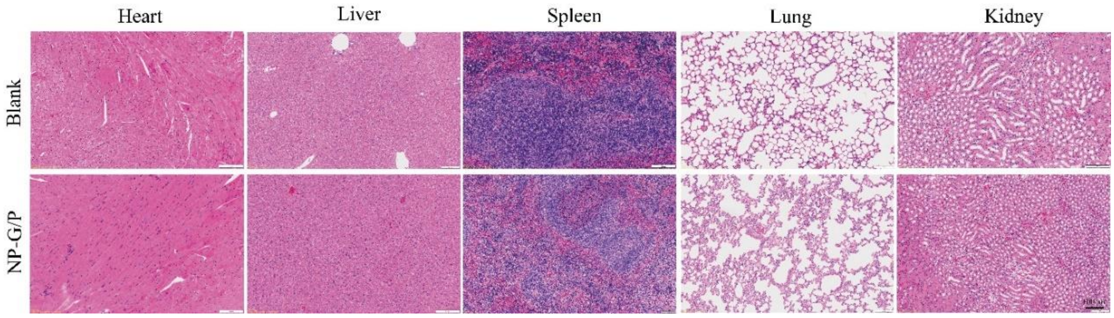

Figure S33. Representative images of H&E-stained slices from major organs in healthy mice after different treatments.

Table S1. Swiss target prediction of GA

Target	Common name	Target Class	Probability*	Known actives (3D/2D)
Inhibitor of nuclear factor kappa B kinase beta subunit	IKBKB	Kinase	1	11/4
Thioredoxin	TXN	Unclassified protein	1	1/1
Thioredoxin, mitochondrial	TXN2	Unclassified protein	1	1/1
Induced myeloid leukemia cell differentiation protein Mcl-1	MCL1	Other cytosolic protein	1	27/1
Caspase-1	CASP1	Protease	0.035898205	257/0
Signal transducer and activator of transcription 3	STAT3	Transcription factor	0.035898205	31/0
Signal transducer and activator of transcription 5B	STAT5B	Transcription factor	0.035898205	9/0
Type-1 angiotensin II receptor (by homology)	AGTR1	Family A G protein-coupled receptor	0.035898205	143/0
Chymase	CMA1	Protease	0.035898205	46/0
Beta-secretase 1	BACE1	Protease	0.035898205	97/0
Steroid 5-alpha-reductase 1	SRD5A1	Oxidoreductase	0.035898205	6/0
Steroid 5-alpha-reductase 2	SRD5A2	Oxidoreductase	0.035898205	8/0
Metabotropic glutamate receptor 2 (by homology)	GRM2	Family C G protein-coupled receptor	0.035898205	36/0
Leukocyte elastase	ELANE	Protease	0.035898205	43/0
Sphingosine 1-phosphate receptor Edg-5	S1PR2	Family A G protein-coupled receptor	0.035898205	20/0
Integrin alpha-4/beta-1	ITGB1 ITGA4	Membrane receptor	0.035898205	412/0
Prostanoid EP1 receptor (by homology)	PTGER1	Family A G protein-coupled receptor	0.035898205	47/0
Adenosine A2a receptor	ADORA2A	Family A G protein-coupled receptor	0.035898205	26/0
Adenosine A2b receptor	ADORA2B	Family A G protein-coupled receptor	0.035898205	11/0
Renin	REN	Protease	0.035898205	149/0
Cysteinyl leukotriene receptor 1	CYSLTR1	Family A G protein-coupled receptor	0.035898205	34/0
Ileal bile acid transporter	SLC10A2	Electrochemical transporter	0.035898205	26/0
G protein-coupled receptor 44	PTGDR2	Family A G protein-coupled	0.035898205	264/0

		receptor		
Inosine-5'-monophosphate dehydrogenase 1	IMPDH1	Oxidoreductase	0.035898205	15/0
Thymidylate synthase (by homology)	TYMS	Transferase	0.035898205	49/0
Signal transducer and activator of transcription 1-alpha/beta	STAT1	Transcription factor	0.035898205	2/0
Serine/threonine-protein kinase Aurora-B	AURKB	Kinase	0.035898205	30/0
Inhibitor of nuclear factor kappa B kinase epsilon subunit	IKBKE	Kinase	0.035898205	5/0
Serine/threonine-protein kinase TBK1	TBK1	Kinase	0.035898205	5/0
Tyrosinase	TYR	Oxidoreductase	0.035898205	4/0
Protein farnesyltransferase	FNTA FNTB	Enzyme	0.035898205	150/0
Cathepsin D	CTSD	Protease	0.035898205	17/0
Motilin receptor	MLNR	Family A G protein-coupled receptor	0.035898205	3/0
Caspase-3	CASP3	Protease	0.035898205	163/0
5-lipoxygenase activating protein	ALOX5AP	Other cytosolic protein	0.035898205	140/0
Caspase-2	CASP2	Protease	0.035898205	22/0
Bile acid receptor FXR	NR1H4	Nuclear receptor	0.035898205	49/0
Receptor-type tyrosine-protein phosphatase S	PTPRS	Phosphatase	0.035898205	3/0
Coagulation factor XI	F11	Protease	0.035898205	32/0
T-cell protein-tyrosine phosphatase	PTPN2	Phosphatase	0.035898205	43/0
FK506-binding protein 1A	FKBP1A	Isomerase	0.035898205	86/0
Peptidyl-prolyl cis-trans isomerase FKBP5	FKBP5	Enzyme	0.035898205	19/0
Calcitonin gene-related peptide type 1 receptor	CALCRL	Family B G protein-coupled receptor	0.035898205	3/0
FK506 binding protein 4	FKBP4	Enzyme	0.035898205	11/0
Angiotensin II receptor	AGTR2	Family A G protein-coupled receptor	0.035898205	38/0
Signal transducer and activator of	STAT5A	Transcription factor	0.035898205	1/0

transcription 5A				
TRAIL receptor-1	TNFRSF10A	Membrane receptor	0.035898205	1/0
MAP kinase p38 alpha	MAPK14	Kinase	0.035898205	42/0
Cholecystokinin A receptor	CCKAR	Family A G protein-coupled receptor	0.035898205	63/0
Purinergic receptor P2Y12	P2RY12	Family A G protein-coupled receptor	0.035898205	247/0
Cholecystokinin B receptor	CCKBR	Family A G protein-coupled receptor	0.035898205	479/0
Neprilysin (by homology)	MME	Protease	0.035898205	95/0
Pepsinogen C (by homology)	PGC	Protease	0.035898205	3/0
Protein kinase C beta	PRKCB	Kinase	0.035898205	5/0
Chromobox protein homolog 7	CBX7	Reader	0.035898205	7/0
Integrin alpha-V/beta-3	ITGAV ITGB3	Membrane receptor	0.035898205	504/0
Phosphodiesterase 5A	PDE5A	Phosphodiesterase	0.035898205	59/0
Membrane-associated guanylate kinase-related 3	MAGI3	Enzyme	0.035898205	5/0
Egl nine homolog 1	EGLN1	Oxidoreductase	0.035898205	9/0
Angiotensin-converting enzyme (by homology)	ACE	Protease	0.035898205	82/0
Vitamin D receptor	VDR	Nuclear receptor	0.035898205	23/0
Glucagon receptor	GCGR	Family B G protein-coupled receptor	0.035898205	135/0
Plasminogen activator inhibitor-1	SERPINE1	Secreted protein	0.035898205	23/0
Transient receptor potential cation channel subfamily M member 8	TRPM8	Voltage-gated ion channel	0	37/0
Thrombin	F2	Protease	0	76/0
Thromboxane A2 receptor	TBXA2R	Family A G protein-coupled receptor	0	125/0
Neurokinin 1 receptor	TACR1	Family A G protein-coupled receptor	0	9/0
Cytosolic phospholipase A2	PLA2G4A	Enzyme	0	91/0
Calpain 1	CAPN1	Protease	0	12/0
Leukocyte adhesion glycoprotein LFA-1 alpha	ITGAL	Adhesion	0	17/0
Histone deacetylase	HDAC1	Eraser	0	30/0

1				
Caspase-8	CASP8	Protease	0	75/0
G-protein coupled receptor kinase 2	GRK2	Kinase	0	1/0
Calcium sensing receptor	CASR	Family C G protein-coupled receptor	0	24/0
Plectin	PLEC	Unclassified protein	0	5/0
Vasopressin V2 receptor	AVPR2	Family A G protein-coupled receptor	0	22/0
Vasopressin V1a receptor	AVPR1A	Family A G protein-coupled receptor	0	16/0
Epoxide hydratase	EPHX2	Protease	0	33/0
HLA class II histocompatibility antigen DRB3-1	HLA-DRB3	Surface antigen	0	8/0
Cathepsin (B and K)	CTSB	Protease	0	30/0
Nuclear receptor ROR-gamma	RORC	Nuclear receptor	0	3/0
Carbonic anhydrase II	CA2	Lyase	0	48/0
Integrin alpha-4/beta-7	ITGB7 ITGA4	Membrane receptor	0	123/0
Aldo-keto-reductase family 1 member C3	AKR1C3	Enzyme	0	16/0
Histone deacetylase 3	HDAC3	Eraser	0	12/0
Serotonin 4 (5-HT4) receptor	HTR4	Family A G protein-coupled receptor	0	7/0
Histone deacetylase 2	HDAC2	Eraser	0	9/0
Integrin alpha-5/beta-1	ITGB1 ITGA5	Membrane receptor	0	54/0
Integrin alpha-V/beta-5	ITGB5 ITGAV	Membrane receptor	0	70/0
Histone deacetylase 8	HDAC8	Eraser	0	10/0
Histone deacetylase 11	HDAC11	Eraser	0	9/0
Endothelin-converting enzyme 1	ECE1	Protease	0	77/0
Histone deacetylase 10	HDAC10	Eraser	0	9/0
Cyclin-dependent kinase 5/CDK5 activator 1	CDK5R1 CDK5	Kinase	0	28/0
Lysosomal Pro-X carboxypeptidase	PRCP	Protease	0	1/0
Protein phosphatase 2A regulatory	PTPA	Phosphatase	0	3/0

subunit B'				
Thrombopoietin receptor	MPL	Membrane receptor	0	6/0
ADAMTS5	ADAMTS5	Protease	0	12/0
ADAMTS4	ADAMTS4	Protease	0	16/0
HERG	KCNH2	Voltage-gated ion channel	0	27/0

Table S2. Relevant parameters of the amino acids binding between TRX and GA.

**CurPocket ID**	**Vina score**	**Cavity volume (A^3)**	**Center (x,y,z)**	**Docking size (x,y,z)**	
C4	-8.1	190	48,7,63	24,24,24	Chain A: HIS-2 GLY-1 SER0 THR1 PHE3 ASP13 ARG14 ASN17 SER18 GLU19 THR20 HIS49 GLY50 LYS51 VAL53
C1	-7.8	15970	35,17,37	35,34,35	Chain A: GLU70 SER72 ALA73; Chain B: GLY32 PRO33 VAL74 PRO75 VAL90 GLY91 ILE92; Chain E: VAL85 VAL86 ASP87 LYS88 PHE89 VAL90 LYS93 GLN97 ALA100 PHE101 LYS104
C5	-6.7	189	44,21,9	24,24,24	Chain B: GLU19 PRO21 GLY50 LYS51 LYS81 ASN82 LEU105 ILE106 GLY107; Chain C: ILE36 PRO39 ARG40 LYS43 GLU95
C3	-5.8	220	34,13,67	24,24,24	Chain A: GLU19 THR20 PRO21 GLY50 LYS51 LYS81 ASN82 LEU105 ILE106 GLY107; Chain F: ILE36 PRO39 ARG40 LYS43 GLU95
C2	-5.7	261	9,2,31	24,24,24	Chain E: HIS-2 THR1 SER18 GLU19 THR20 PRO21 HIS49 GLY50 LYS51 VAL52 VAL53 LYS81 ILE106

Table S3. Pharmacokinetic parameters of NP and NP-G/P in mice

Parameters	NP	NP-G/P
**$t_{1/2}$ (h)**	11.38±1.31	10.97±0.13
**$AUC_{0-\infty}$ (%ID/mL*h)**	3.01±0.10	3.32±0.21
**Cl (L/h/kg)**	8.41±0.27	7.55±0.48
